# Causal Relationship of Susceptibility Genes to Ischemic Stroke: Comparison to Ischemic Heart Disease and Biochemical Determinants

**DOI:** 10.1371/journal.pone.0009136

**Published:** 2010-02-09

**Authors:** Paul Bentley, George Peck, Liam Smeeth, John Whittaker, Pankaj Sharma

**Affiliations:** 1 Imperial College Cerebrovascular Research Unit, Clinical Neurosciences, Charing Cross Hospital, Imperial College London, London, United Kingdom; 2 Department of Epidemiology and Population Health, London School of Hygiene & Tropical Medicine, London, United Kingdom; Baylor College of Medicine, United States of America

## Abstract

Interrelationships between genetic and biochemical factors underlying ischemic stroke and ischemic heart disease are poorly understood. We: 1) undertook the most comprehensive meta-analysis of genetic polymorphisms in ischemic stroke to date; 2) compared genetic determinants of ischemic stroke with those of ischemic heart disease, and 3) compared effect sizes of gene-stroke associations with those predicted from independent biochemical data using a mendelian randomization strategy. Electronic databases were searched up to January 2009. We identified: 1) 187 ischemic stroke studies (37,481 cases; 95,322 controls) interrogating 43 polymorphisms in 29 genes; 2) 13 meta-analyses testing equivalent polymorphisms in ischemic heart disease; and 3) for the top five gene-stroke associations, 146 studies (65,703 subjects) describing equivalent gene-biochemical relationships, and 28 studies (46,928 subjects) describing biochemical-stroke relationships. Meta-analyses demonstrated positive associations with ischemic stroke for factor V Leiden Gln506, ACE I/D, MTHFR C677T, prothrombin G20210A, PAI-1 5G allele and glycoprotein IIIa Leu33Pro polymorphisms (ORs: 1.11 – 1.60). Most genetic associations show congruent levels of risk comparing ischemic stroke with ischemic heart disease, but three genes—glycoprotein IIIa, PAI-1 and angiotensinogen—show significant dissociations. The magnitudes of stroke risk observed for factor V Leiden, ACE, MTHFR and prothrombin, but not PAI-1, polymorphisms, are consistent with risks associated with equivalent changes in activated protein C resistance, ACE activity, homocysteine, prothrombin, and PAI-1 levels, respectively. Our results demonstrate causal relationships for four of the most robust genes associated with stroke while also showing that PAI-1 4G/5G polymorphism influences cardiovascular risk via a mechanism not simply related to plasma levels of PAI-1 (or tPA) alone.

## Introduction

Stroke is one of the leading causes of death, disability, and health finance cost in both developed and developing world countries [1]. Understanding the genetic contributions to ischemic stroke is important not only so as to explain, or predict, the minority of cases that occur in the absence of well-established risk factors, such as smoking, hypertension and diabetes [2], but also to account for wide variability of stroke incidence within individuals who do harbour these common, acquired risk-factors [3]. Moreover, appreciating the biochemical basis for risk-associated genes can motivate novel therapeutic strategies, including pharmacogenomics [4].

As the cumulative number of studies reporting positive genetic associations with stroke increases, the main challenges are deciding which associations are reliable and robust, and then deciphering the role of putative gene effects in terms of causation [5]. The present study attempts to address these issues by firstly, presenting the most comprehensive meta-analysis to date of all candidate genetic polymorphisms associated with ischemic stroke. Secondly, we relate these observed gene effect sizes with those predicted from pathophysiologically-related studies.

Since many of the candidate genes tested for an association with ischemic stroke have originated from studies in ischemic heart disease, and given overlapping pathophysiologies of these two diseases [6], it is meaningful to investigate whether specific genetic polymorphisms associate with clinical arteriopathic syndromes in general, e.g. due to a tendency to stiffen arteries [7], or whether certain genes exert organ-specific effects [8]–[10]. Furthermore, where positive associations do exist between genes and stroke it is critical to validate whether these effects are consistent with the risks attributed to their putative biological intermediates. For example, if a stroke-associated gene is also associated with a prothrombotic tendency, then does the degree of thrombophilia imparted by the genotype-in-question associate with a similar degree of risk of stroke, using independent data sets? We attempted to answer this question for all robust positive gene associations using a method based upon mendelian randomization [11].

## Results

### Ischemic Stroke Candidate Gene Meta-Analysis

We identified 187 candidate genetic polymorphism case-control studies (References S1), incorporating 37,481 ischemic stroke cases and 95,322 controls that fulfilled the inclusion criteria. Between them, 43 polymorphisms were interrogated in 29 genes, with the mean number of studies per candidate polymorphism being 6.6 (95% CIs 4.4 – 8.8). For 23 out of the 43 candidate polymorphisms (53%), the combined studies comprised >1000 cases (and >1000 controls) in aggregate. It is these that are focused on in the rest of the results. Note that these represent 16 out of 29 candidate *genes*, because for several genes more than one polymorphism was tested; PDE 4D (6 SNPs), angiotensinogen (2 SNPs) and HFE (2 SNPs) being those for which there are >1000 pooled cases. Eighteen polymorphisms (42% of the total; representing 11 genes) were investigated by at least one study comprising a total sample size of >1000.

**Figure 1:**
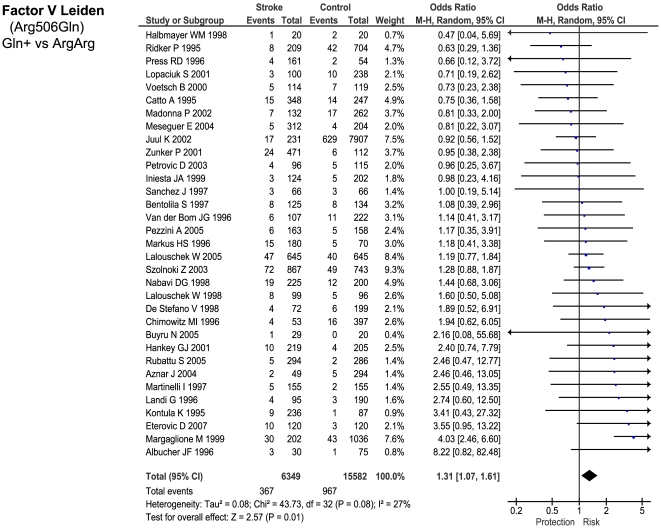
Forest plots showing positive associations of ischemic stroke with the following genetic polymorphisms: Factor V Leiden Arg506Gln.

Of the 23 genetic polymorphisms candidates tested in >1000 cases, six polymorphisms in six genes were found to show an overall significant effect, with no significant between-study heterogeneity (Figures 1–7). These were, in order of case-numbers: factor V Leiden Gln506, angiotensin converting enzyme (ACE) I/D, methylene tetrahydrofolate reductase (MTHFR) C677T, prothrombin G20210A, plasminogen activator inhibitor-1 5G allele and glycoprotein IIIa Leu33Pro. The summary ORs for these genes ranged from 1.15 (95% CI: 1.06 – 1.25) for ACE I/D, to 1.60 (95% CI: 1.28 – 2.00) for prothrombin G20210A. The corresponding population attributable risks for the genes listed above are, respectively: 1.8%, 3.9%, 3.1%, 1.9%, 11.2% and 5.8% (total: 27.5%). The remainder 17 polymorphisms that were tested in >1000 pooled cases failed to demonstrate association with ischemic stroke (Figure 7; Figures S1). Within this group, ten polymorphisms showed between-study heterogeneity (p<0.05).

**Figure 2 pone-0009136-g002:**
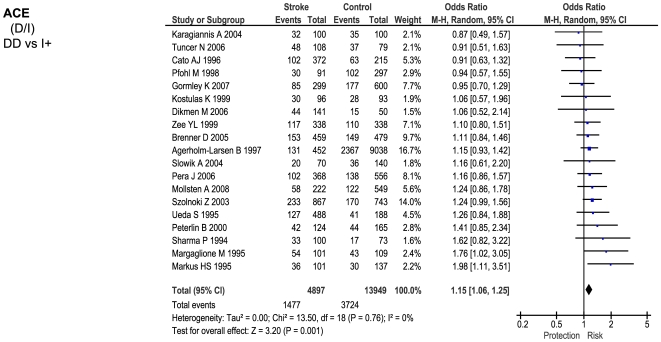
Forest plots showing positive associations of ischemic stroke with the following genetic polymorphisms: ACE D/I.

**Figure 3 pone-0009136-g003:**
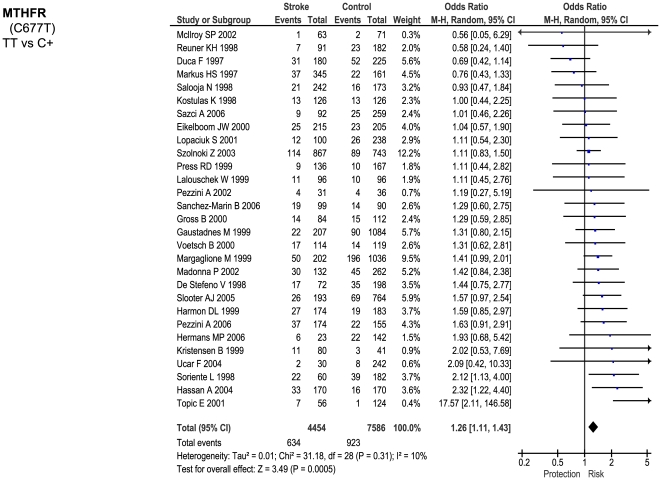
Forest plots showing positive associations of ischemic stroke with the following genetic polymorphisms: MTHFR C677T.

**Figure 4 pone-0009136-g004:**
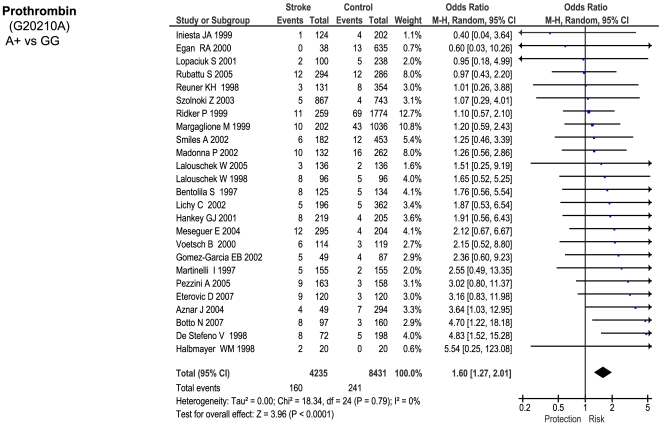
Forest plots showing positive associations of ischemic stroke with the following genetic polymorphisms: prothrombin G20210A.

**Figure 5 pone-0009136-g005:**
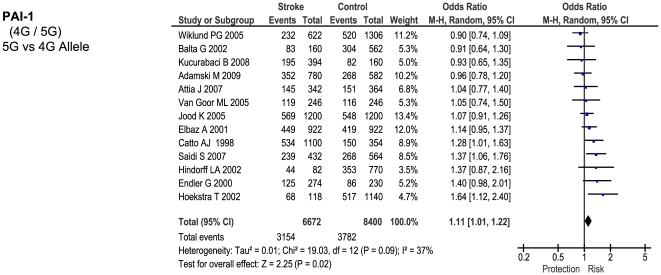
Forest plots showing positive associations of ischemic stroke with the following genetic polymorphisms: PAI 5G allele.

**Figure 6 pone-0009136-g006:**
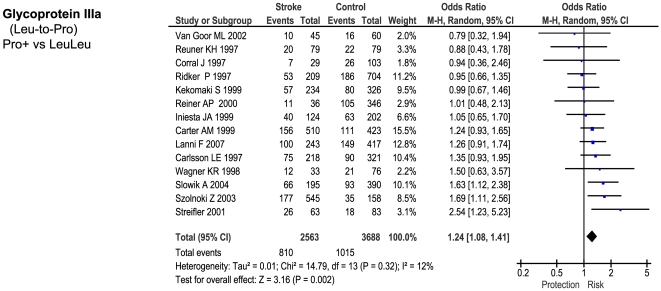
Forest plots showing positive associations of ischemic stroke with the following genetic polymorphisms: glycoprotein IIIa Leu33Pro.

**Figure 7 pone-0009136-g007:**
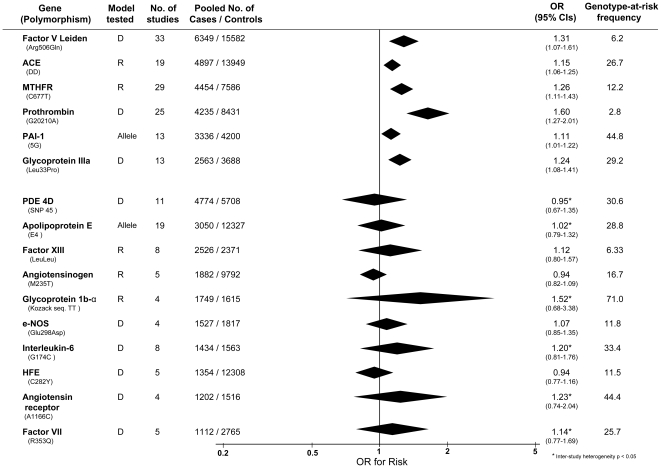
Summary of meta-analyses testing associations of candidate genetic polymorphisms with ischemic stroke. Table reports genetic model tested (D – dominant; R – recessive); numbers of studies; numbers of pooled cases and controls; ORs with 95% confidence intervals, and at-risk genotype frequency.

Of the 20 candidate polymorphisms that were tested in <1000 cases, four were found to show positive associations: factor VII R353Q (793 cases), protein Z G79A (741 cases), glycoprotein 1b-alpha Met-Thr (564 cases), and intercellular adhesion molecule-1 E469K (356 cases).

For all of the above associations there was no publication bias towards smaller studies, as indicated by non-significant (p>0.1) regression intercepts in Egger funnel-plots of effect size against sample variance [12].

**Figure 8 pone-0009136-g008:**
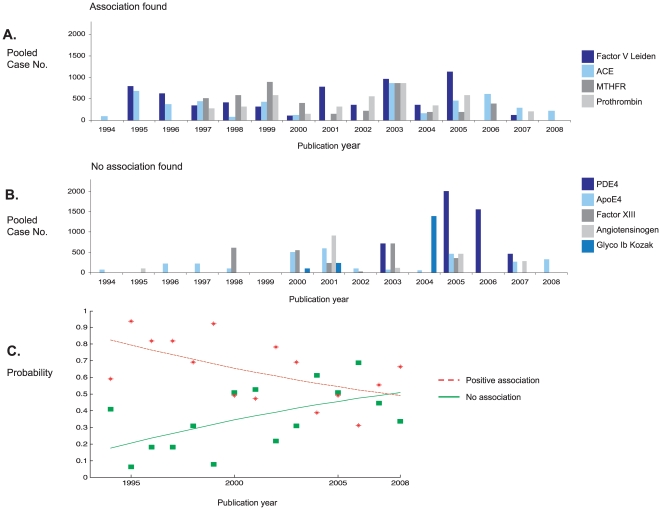
Trends in success of candidate gene approach. **A**: Numbers of pooled cases published over time testing for polymorphisms positively associated with stroke according to the present meta-analyses. **B**: As for A, but for polymorphisms found to show no stroke association according to the present meta-analysis. **C**: Changes in time of probability that cases were tested for polymorphism subsequently found to show association (red) or no association (green) with stroke.

In order to evaluate the relative efficiency of the candidate gene method over time, we divided studies according to whether our meta-analysis either identified, or failed to identify, a significant association, and plotted pooled case numbers for each of the largest polymorphisms against publication year (Figure 8 A and B).This shows that the during the first decade of published studies (1993 – 2003) candidate polymorphisms were predominantly those found to be associated with stroke (according to our meta-analysis), whereas more recently (2004+), an increasing number of studied cases are for polymorphisms that show no association after pooling. Such declining success of candidate-gene studies is also seen by plotting the probability that cases were tested for polymorphisms that were found after meta-analysis to be associated, rather than unassociated, over time (Figure 8 C). We note that there were no correlations between OR magnitude, or study size, and publication date, when analyzing polymorphisms individually or grouping as a whole (all p<0.1).

### Comparison with Ischemic Heart Disease

**Figure 9 pone-0009136-g009:**
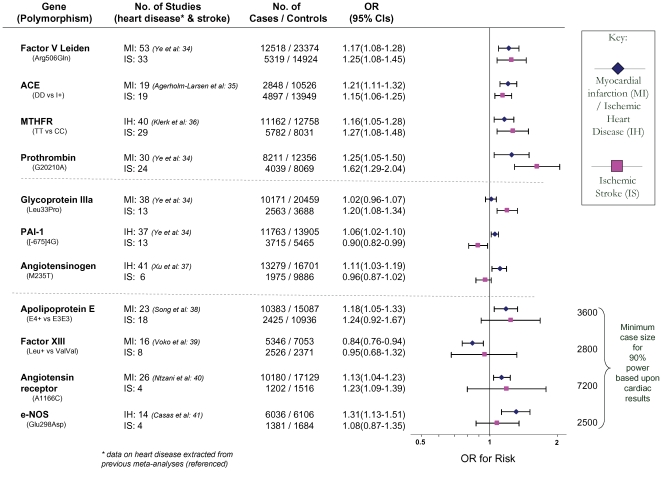
Risk of genetic polymorphisms compared between ischemic stroke (IS) and ischemic heart disease (IH) or myocardial infarction (MI). Contrasts represent per-allele effects except where indicated. Polymorphisms are grouped into those showing consistent associations for both types of disease; those showing dissociated effects; and those showing a significant risk of heart disease but not stroke. For the latter the minimum sample numbers estimated to achieve 90% power based upon each polymorphism's effect size for ischemic heart disease are shown.

Meta-analyses in myocardial infarction and/or ischemic heart disease were found [13]–[22] for thirteen of the genetic polymorphisms tested in ischemic stroke for which there were >1000 pooled cases. Comparing odds ratios for each genetic polymorphism between ischemic cardiac disease and ischemic stroke identified four profiles (Figure 9):

1. Polymorphisms associated with risk of *both* ischemic heart disease and stroke: factor V Leiden Gln506, ACE I/D, MTHFR C677T, prothrombin G20210A. The 95% confidence intervals (CIs) for ORs of ischemic stroke and cardiac disease overlapped for all of these gene variants.
2. Polymorphisms associated with risk of *either* ischemic stroke *or* myocardial ischemia but not the other disease type – i.e. dissociations: glycoprotein IIIa Leu33Pro conferring a risk for stroke, but not ischemic heart disease; PAI-1 4G- versus-5G associated positively with cardiac disease, but negatively with stroke, and angiotensinogen M235T posing a risk for coronary stenosis, but a trend for protection against stroke. The 95% CIs for risk for all three polymorphisms were non-overlapping comparing ischemic stroke with ischemic heart disease, although restricting cardiac datasets to those in which Caucasian ethnicity was definitely specified results in marginal overlap for glycoprotein IIIa Leu33Pro (ischemic heart disease OR: 0.99 – 1.09) and angiotensinogen M235T (coronary stenosis OR: 1.0 – 1.16).
3. Polymorphisms associated with myocardial ischemia but not ischemic stroke, but where confidence intervals clearly overlapped. In these cases, we calculated the minimum case number required for 90% power (and α = 0.05) assuming the same effect size as in ischemic heart disease. This showed that in all cases an inadequate sample size in stroke may account for the lack of significance here (Figure 9; third section, final column).
4. Polymorphisms not associated with either ischemic stroke or heart disease yet tested in >1000 cases of each disease viz. interleukin-6 G174C [21] and HFE C282Y [22].

### Comparison with Biochemically-Predicted Risk

For each of the stroke-associated genetic polymorphisms identified by our primary meta-analysis we searched for: 1) studies providing differential measurements of a biochemical marker - i.e. an intermediate phenotype, IP - related to the genotypic variants of interest, among subjects *without* cardiovascular disease, and 2) studies providing an estimate of ischemic stroke risk based upon incremental change in the same IP. Such studies were available for the following polymorphism – IP pairs: factor V Leiden - activated protein C resistance, ACE D/I - ACE activity, MTHFR - homocysteine levels, prothrombin G20210A - prothrombin levels, and PAI-1 4G/5G - PAI-1 levels.

**Figure 10:**
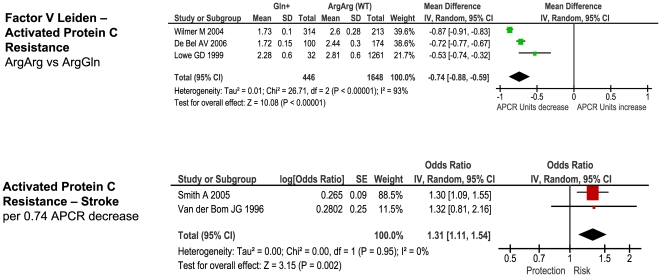
Forest plots showing quantitative relationship between genetic polymorphisms and associated biochemical variables for: Factor V Leiden and activated Protein C resistance ratio. Additional forest plots are shown in Figures 10, and 13– [Fig pone-0009136-g014] 15 that relate set changes in biochemical variables (determined from the first set of meta-analyses within each figure) with risk of stroke. For MTHFR and ACE this relationship is determined from a single study each.

The number of studies providing differential IP levels according to genotype ranged between 3 and 70, incorporating between 843 and 46,743 healthy subjects, for each genotype of interest. We performed meta-analyses that pooled these biomarker level differences for each of the six genotype – IP pairs (Figures 10 – [Fig pone-0009136-g011] [Fig pone-0009136-g012] [Fig pone-0009136-g013] [Fig pone-0009136-g014] 15), with the weighted mean differences, and 95% CIs, summarised in Figure 16 (2^nd^ column).

**Figure 11 pone-0009136-g011:**
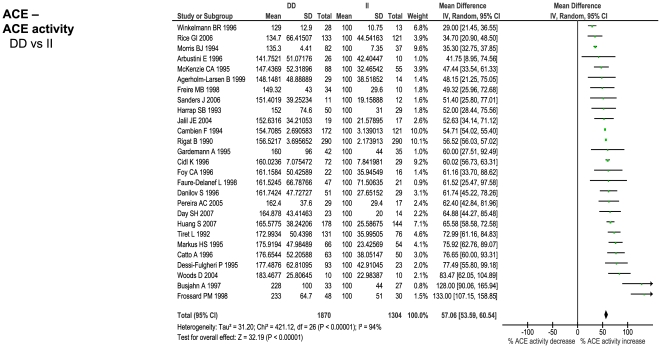
Forest plots showing quantitative relationship between genetic polymorphisms and associated biochemical variables for: ACE D/I and ACE activity. Additional forest plots are shown in Figures 10, and 13– [Fig pone-0009136-g014] 15 that relate set changes in biochemical variables (determined from the first set of meta-analyses within each figure) with risk of stroke. For MTHFR and ACE this relationship is determined from a single study each.

**Figure 12 pone-0009136-g012:**
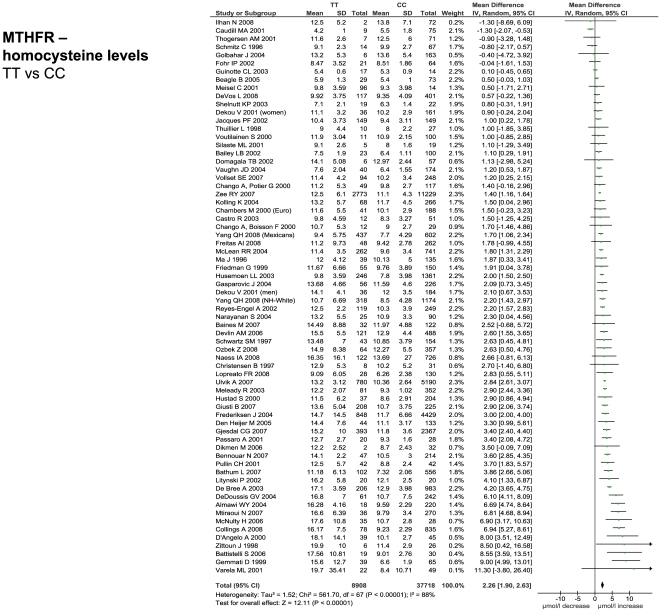
Forest plots showing quantitative relationship between genetic polymorphisms and associated biochemical variables for: MTHFR and homocysteine levels. Additional forest plots are shown in Figures 10, and 13– [Fig pone-0009136-g014] 15 that relate set changes in biochemical variables (determined from the first set of meta-analyses within each figure) with risk of stroke. For MTHFR and ACE this relationship is determined from a single study each.

**Figure 13 pone-0009136-g013:**
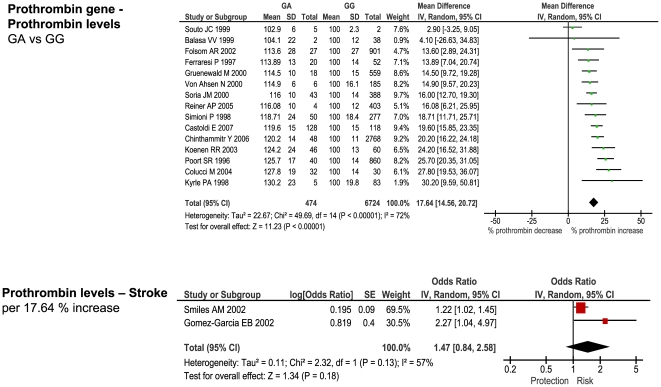
Forest plots showing quantitative relationship between genetic polymorphisms and associated biochemical variables for: Prothrombin G20210A and prothrombin levels. Additional forest plots are shown in Figures 10, and 13– [Fig pone-0009136-g014] 15 that relate set changes in biochemical variables (determined from the first set of meta-analyses within each figure) with risk of stroke. For MTHFR and ACE this relationship is determined from a single study each.

**Figure 14 pone-0009136-g014:**
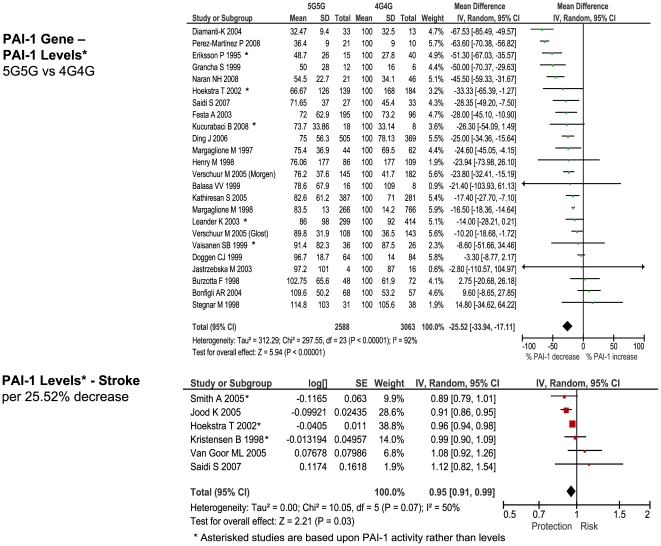
Forest plots showing quantitative relationship between genetic polymorphisms and associated biochemical variables for: PAI-1 5G/4G and PAI-1 levels. Additional forest plots are shown in Figures 10, and 13– [Fig pone-0009136-g014] 15 that relate set changes in biochemical variables (determined from the first set of meta-analyses within each figure) with risk of stroke. For MTHFR and ACE this relationship is determined from a single study each.

**Figure 15:**
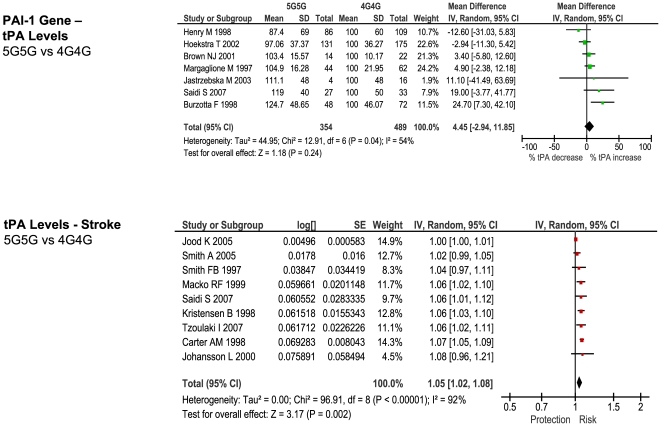
Forest plots showing quantitative relationship between genetic polymorphisms and associated biochemical variables for: PAI-1 5G/4G and tPA levels. Additional forest plots are shown in Figures 10, and 13– [Fig pone-0009136-g014] 15 that relate set changes in biochemical variables (determined from the first set of meta-analyses within each figure) with risk of stroke. For MTHFR and ACE this relationship is determined from a single study each.

**Figure 16:**
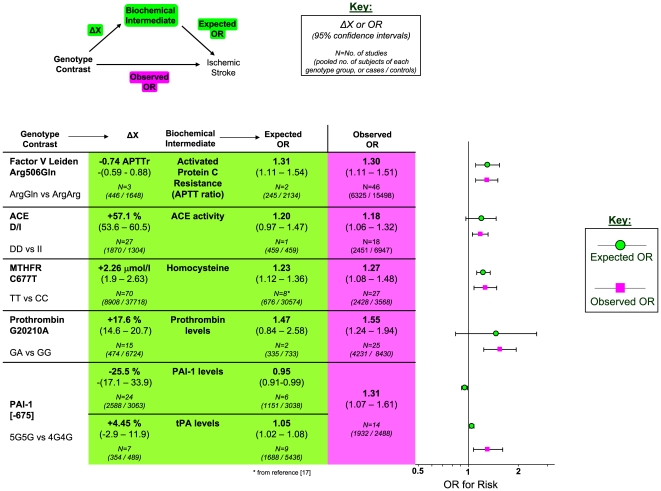
Comparison of estimated risk with observed risk for ischemic stroke-associated genetic polymorphisms. Estimated ORs (green) are calculated from 1) meta-analyses relating risk-genotype with biochemical variation (ΔX), and 2) single studies or meta-analyses relating biochemical variation with stroke risk (scaled log-linearly). Observed ORs are derived from meta-analyses of the equivalent genotype contrast using the current datasets.

The number of studies providing estimates of ischemic stroke risk relative to incremental changes in the above IPs ranged between 1 and 9, incorporating between 245 and 1688 cases, and 459 and 30574 controls. Where more than one study existed that provided an estimate of IP – stroke risk, we performed a meta-analysis that combined these ORs (Figures 10 and 13 – [Fig pone-0009136-g014] 15; lower forest plots), whilst first adjusting each value to the pooled estimate of biomarker level change for the genotype comparison of interest (see above), assuming a log-linear relationship. In the case of ACE activity and homocysteine levels, a single estimate of biomarker - stroke risk was extracted from a single study [23], and previous meta-analysis [24], respectively, and then scaled in the same way. The results of these calculations for expected risk of the genotype comparisons of interest (Figure 16, 4^th^ column) were then compared with the observed risk for each of the same genotype contrasts, based upon data used in the first part of this paper (5^th^ column).

As an example, Factor V Leiden 506 mutation ArgGln, relative to wild-type ArgArg, is associated with a weighted mean average of 0.74 APTT ratio decrease in the activated protein C resistance test in healthy subjects (Figure 10, upper). Independent data from ischemic stroke case – control studies suggest that a 0.74 decrease in APTT ratio using the same test is associated with a risk odds-ratio of 1.31 (1.11 – 1.54) (Figure 10, lower). This estimated OR is very close to the observed OR of 1.30 (1.11 – 1.51) noted from our earlier meta-analysis (Figure 1).

For each of the gene – biomarker pairs (Figure 16), the mean level of expected risk fell close to, and certainly within the 95% confidence intervals, the observed risk, with the exception of PAI-1. For this latter pairing, the expected risk of the genotype comparison 5G5G vs 4G4G based upon PAI-1 levels was significantly lower than that observed. This arose because whilst PAI-1 levels are found to be higher in stroke than in controls, the 5G5G genotype – that itself is associated with elevated stroke risk relative to 4G4G - was associated with a ∼25% reduction in PAI-1 levels relative to 4G4G. This result was unaffected by whether PAI-1 levels were derived from antigen concentration or enzyme activity data. We also estimated expected stroke risk on the basis of tPA levels, and its relationship with the PAI-1 4G/5G genotype. The 5G5G relative to 4G4G genotype was associated with a slight, albeit non-significant, increase in tPA levels, which itself is independently associated with stroke. However, the expected stroke risk using tPA data was also less than that observed.

## Discussion

The current study takes a meta-analytic approach to identify the totality of candidate genetic polymorphisms reliably associated with ischemic stroke. Furthermore, we show how these results compare with the same polymorphisms in ischemic cardiac disease, and test their causal relationship with likely biochemical intermediaries. These points are discussed in turn.

### Meta-Analysis of Genetic Polymorphisms in Ischemic Stroke

Since our last comprehensive meta-analysis of gene effects in ischemic stroke [25], the size of the pooled study sample from which we were able to extract genetic polymorphism frequency data has more than doubled (from ∼18000 to ∼37000 cases), as has the number of genetic polymorphisms in which more than 1000 cases have been tested for each (from 8 to 23). In order to increase reliability [5] we confined our results to gene variants studied in over 1000 patients, and to studies where ischemic stroke was confirmed radiologically, and that were based predominantly in Caucasian adults. We discounted studies where there was publication bias, and applied random-effects models to allow for between-study heterogeneity. After applying these criteria we found positive associations with polymorphisms in the following six genes: Factor V Leiden, ACE, MTHFR, prothrombin, PAI-1, and glycoprotein-III.

As well as identifying two further genetic associations not identified reliably in our earlier meta-analysis [25], our study supports the validity of the meta-analysis technique by finding very similar effect sizes for the four positive associations identified previously in the face of an increase in patient numbers by approximately 50%. There was no reduction in effect size comparing the current results with those from five years earlier [25] as is sometimes observed [26]. A difference in our result for the PAI-1 polymorphism relative to a recent meta-analysis [27] can be explained by our more rigorous inclusion criteria that restricted data to Caucasian-predominant populations [28], and confirmed cerebral infarcts [29].

Whilst the effect size of each positive gene association was small (odd ratios of 1.11 to 1.60), the overall contribution that genetic factors make towards stroke is likely to be relatively large given the frequency of these risk variants in the general population (from 3 to 45% each). The sum of the population attributable risks across all the gene associations identified here was ∼30%. These results are in keeping with models of common complex diseases in which relatively small numbers of common polymorphisms, each with only small hazard ratios, can account for large proportions of population attributable risk [30]. By contrast, certain well-described single-gene mutations may confer a high relative risk of stroke, e.g. CADASIL, but contribute very little to overall stroke occurrence by virtue of their rarity.

It is likely that future discovery of disease-associated genes will rest increasingly with genome-wide association studies (GWAS), rather than candidate-gene strategies [9]. For example, the recent finding from genome-wide searches that polymorphisms on chromosome 9p are associated with myocardial infarction, has prompted preliminary testing of the same polymorphisms in ischemic stroke [31], [32]. In fact, we found that an increasing proportion of recruited cases to candidate-gene studies over time are testing genetic polymorphisms that, after pooling over large numbers, fail to show associations. This is unlikely to reflect a publication bias towards positive results amongst earlier publications since we found no association between effect magnitude, or sample size, and publication date; moreover, the summary ORs for candidate polymorphisms have not changed significantly over five years [25]. In the last two years the overall number of candidate-gene studies, and recruited patients, has fallen that may reflect increasing inefficiency in selecting candidate genes and increased employment of GWAS strategies.

### Comparison of Genetic Effects with Ischemic Heart Disease

Given overlapping pathological substrates for ischemic heart disease and ischemic stroke, as well as a common set of acquired risk factors and treatment strategies [9], we expected a broadly similar set, and relative strength, of genetic associations. For the majority of polymorphisms reported here this is what was found. Polymorphisms were found to impose similar degrees of risk for ischemic heart disease and stroke, or were found to show no associations for both. In some cases, a similar risk was found for both disease types, but significance was only achieved for ischemic heart disease, probably due to inadequate power of pooled stroke studies.

Three genetic polymorphisms tested in both ischemic heart disease and ischemic stroke showed differing effects between diseases that did not seem to arise from inadequate power in either group. We are cautious in our interpretation of these apparent disease ‘dissociations’ as the two sets of meta-analyses are not strictly controlled for confounders such as age, sex, or co-morbidities, and case ascertainment methods differ, as are likely to be the thresholds at which coronary versus cerebrovascular ischemia manifest themselves clinically. Nevertheless, both our meta-analyses in stroke, and those cited in heart disease, are based predominantly in non-selective Caucasian adult populations; required both clinical and confirmatory diagnostic procedures; and checked for publication bias. Furthermore, in the cases of PAI-1 and angiotensinogen polymorphisms, it is difficult to account for how the same genotype could produce *opposite* effects on risk comparing the two disease types on the basis of confounding or bias alone.

Further studies, ideally within the same populations, will be needed to confirm whether these three gene-disease dissociations are real, or whether they reflect methodological differences. The fact that these dissociations relate to proteins in three different physiological systems, viz. platelets (glycoprotein IIIa), clotting (PAI-1) and blood pressure (angiotensinogen) - for which there also polymorphisms showing concordant disease effects, e.g. glycoprotein 1b-alpha, factor V Leiden and ACE, respectively – suggests that differences in risk-profile between ischemic heart disease and stroke cannot be attributed simply to a single pathophysiology e.g. hypertension. Differences between ischemic stroke and heart disease in terms of these three pathophysiologies has support from other sources. For example, ischemic stroke relative to ischemic heart disease has a stronger relationship with hypertension [33], [34], whilst the protective profile of anti-platelet and thrombolytic drugs differs between these two diseases [9], [35]. Genetic influences on ischemia may also differ according to vessel size [10].

### Comparison of Genetic Effects with Biochemical Markers of Risk

For each of the positive gene associations with ischemic stroke that we identified, we performed further analyses using separate data to establish whether the putative biochemical intermediaries of these gene variants are associated with equivalent quantitative levels of risk. The method used here was based upon mendelian randomization in which one starts with an observational association between an environmental (e.g. biochemical) factor and disease, and then secondarily investigates whether a concordant level of risk occurs for a genotype that simulates the environmental factor - thereby making a stronger case for the factor being causative of the disease [36]. In the current paper, we start with positive gene-stroke associations and then subsequently interrogate independent biochemical data with the expectation of finding concordant levels of risk.

For the four strongest positive gene-stroke associations– factor V Leiden, MTHFR, ACE and prothrombin - concordance between observed risk and that predicted from their associated biochemical changes was found. Moreover, the mean levels of predicted and observed risks for these genotypes lay very close to each other. Importantly therefore, we show here, for the first time, that the four genetic variants most reliably associated with ischemic stroke are also associated with biochemical changes that themselves are related to equivalent levels of risk. This concordance both validates our original gene – stroke positive associations, and furthermore, suggests that the risk imparted by each genotype variant is the direct consequences of each gene's understood biochemical actions. The fact that each of these genes exerts biochemical or haematological changes that are measurable systemically (i.e. from venous plasma samples), rather than being specifically cerebrovascularly based, is in keeping with our other finding that these four gene variants exert similar levels of risk on ischemic heart disease as on ischemic stroke. In the case of the concordant relationship between MTHFR genotype and homocysteine in their separate associations with stroke risk, we have replicated our earlier findings in the face of more than a fourfold increase in meta-analysis size [11].

In contrast to concordant gene-biochemical risk estimates observed for the four largest genetic associations, we observed a discordant gene – biochemical relationship for the PAI-1 4G/5G polymorphism. Specifically, the variant 5G5G, relative to 4G4G, was associated with an elevated risk of ischemic stroke, but *decreased* PAI levels. However, in separate case-control studies, stroke - as well as atherothrombosis and ischemic cardiac disease [37], [38] - is associated with *increased* PAI-1 levels. Indeed we saw earlier how in ischemic heart disease, it is the 4G allele - associated with higher PAI-1 levels – that is associated with risk. The expected risk of stroke of the 4G/5G PAI polymorphism was also less than that observed using tPA levels, that are strongly influenced by PAI-1 levels.

There are several possible explanations for the apparent PAI-1 gene-biochemical paradox in the case of stroke. Firstly, it is possible that the PAI-1 5G allele association with stroke is false, e.g. because of reporting bias. However, it is unclear why such a false association should emerge in the opposite direction to that expected from ischemic cardiac disease and PAI-1 level data, and Egger's regression test argues against publication bias here [12]. Secondly, the finding that 5G polymorphism depresses PAI-1 levels over the course of the subject's life may have different pathophysiological implications, e.g. by predisposing to stroke, than the same depression of PAI-1 levels found at a single point in later life, when this is found to be protective [36]. A third possibility is that the PAI-1 genetic 4G/5G polymorphism is associated with a brain-specific factor that influences stroke more strongly than does its actions on PAI-1 levels. This may occur because of linkage disequilibrium with an as-yet unidentified gene [39], or because of pleiotropy [36]. For example, while raised PAI-1 levels in plasma may raise thrombotic risk, raised PAI-1 levels in carotid vessel wall may serve to stabilise atheromatous plaques [40]. If the PAI 4G/5G polymorphism influences both tissue and plasma PAI-1 levels, whilst the latter is also unduly influenced by environmental variables [41], this could explain the paradox. Furthermore, postulating that PAI-1 4G/5G exerts different phenotypic effects on cerebral versus other vascular beds might explain why its influences on ischemic stroke and ischemic heart disease are in opposite directions.

### Conclusion

The current study provides a comprehensive meta-analysis of common genetic polymorphisms associated with ischemic stroke that were identified through the candidate gene approach. The results serve as an important comparator to emerging genome-wide association studies [9], [31], [32], which would be expected to converge upon similar genetic associations to those shown here, as well as to identify genes not previously implicated with cardiovascular disease pathogenesis. Since a major purpose of elucidating genetic influences on stroke (as for any complex disease) is to gain insights into its pathophysiology, we show here how pooled gene association data can be meaningfully compared with separate data relating the same genetic effects with both a pathophysiologically-related disease (here, ischemic cardiac disease) and biochemical intermediaries.

## Methods

### 1: Genetic Polymorphism – Ischemic Stroke Association Meta-Analysis

Electronic databases (Medline; EMBASE; Google Scholar) were searched upto January 1, 2009 for all case-control studies evaluating any candidate genetic polymorphism in ischemic stroke. Search words used were: *cerebrovascular disease*, *brain infarction*, *stroke* and *cerebral ischemia* in combination with *polymorphism*, *genetic*, *mutation*, *genotype* and *genes*. All languages were searched and translated when necessary. Additional studies were sought from references, citations and from the PubMed option ‘Related Articles’, for each identified study.

Inclusion criteria were studies that: 1) employed case-control methods where ischemic stroke was analyzed as a dichotomous trait; both retrospective and prospective cohort designs were included; 2) confirmed the diagnosis of ischemic stroke with neuroimaging, and 3) were based in Caucasian populations, so as to minimise inter-racial heterogeneity [28]. Studies were excluded if: 1) patients were aged under 18 years; 2) only quantitative traits or intermediate phenotypes were being investigated, or 3) genotype frequency was not reported. For duplicate publications, the smaller data set(s) were discarded. For studies with more than one control group, the most appropriate control group was used.

Data were analyzed using software for preparing Cochrane reviews (Review Manager, *v*5; Comprehensive Meta Analysis *v*2.2.023). For each genetic polymorphism for which data were available from at least two studies, a meta-analysis was carried out. For each gene variant, a pooled odds ratio (OR) and 95% confidence intervals were calculated using random-effects [42] models, to estimate association strength. Tests for heterogeneity [43], and publication bias [12] were performed with significance set at p<0.05. Random-effects results are reported throughout. In order to improve reliability, we only present here results for polymorphisms tested in >1000 pooled cases [5]. The proportion of cases in the population that could be attributed to a particular genetic variant (population attributable risk or PAR) was estimated as follows: PAR = 100 × [Prevalence(OR – 1)/(Prevalence (OR – 1)+1)]. The prevalence of exposure was estimated as the genotype frequency among pooled controls.

We also sought to determine temporal trends in the success of the candidate-gene approach by determining the pooled number of cases recruited into studies that tested for polymorphisms that were either associated, or not associated, with stroke according to our meta-analysis, and then plotting this against study publication year. Moreover, for every publication year, we calculated the probability that cases were tested for a polymorphism that demonstrated either a significant, or no, association by our meta-analysis. This analysis is restricted to polymorphisms for which there were >1000 pooled cases.

### 2: Ischemic Stroke – Ischemic Heart Disease Genetic Comparison

For each genetic polymorphism tested in ischemic stroke (with >1000 cases), we searched the literature for the most recent meta-analysis testing for the same polymorphism in myocardial infarction and/or ischemic heart disease, based predominantly in Caucasian populations. For all polymorphisms showing a positive association with ischemic stroke there was an equivalent published meta-analysis in ischemic heart disease/myocardial infarction. For each polymorphism for which a cardiac meta-analysis existed we calculated the pooled random-effects OR of ischemic stroke for the equivalent genotype (or per-allele) comparison using our own meta-analysis.

### 3: Gene – Intermediate Phenotype Comparison

For each positive genetic association identified from the meta-analysis in part 1, we performed a separate analysis that produced an estimate of expected risk based upon genotype – biochemical, and biochemical – stroke, association studies, using the principle of mendelian randomisation [11]. Firstly, we searched the medical literature for two further types of study: 1) those relating each genetic polymorphism with a quantitative measure of the most strongly-associated biochemical or hematological marker - i.e. an intermediate phenotype, IP, for the gene in question - in populations free from cardiovascular disease, and 2) those relating an incremental change of the same IP with risk of ischemic stroke. For example, for the ACE polymorphism we searched for: 1) (*ACE* OR *angiotensin converting enzyme*) AND (*gene* OR *genetic* OR *genotype* OR *polymorphism* OR *mutation*), in combination with (*ACE* OR *angiotensin converting enzyme*) AND (*activity* OR *level*); and 2) (*ACE* OR *angiotensin converting enzyme*) AND (*activity* OR *level*) in conjunction with (*cerebrovascular disease* OR *brain infarction* OR *stroke* OR *cerebral ischemia*). As for part 1, we restricted studies to those based in adults from predominantly Caucasian populations.

From these two sets of studies we performed two types of analyses: 1) From the first set of studies we extracted from each the difference in levels (or biological activity) of the IP between the two homozygous variants, or between the wild-type and heterozygous variants in cases of dominant polymorphisms. These values were entered into a meta-analysis to obtain a weighted mean average using a random-effects model. For those IPs where different measurement units had been employed between studies (viz. ACE activity; prothrombin levels, plasminogen-activator inhibitor-1 levels and tissue plasminogen activator levels) we calculated the percentage change for the rarer, relative to the commoner, genotype. 2) From the second set of studies we obtained an OR of ischemic stroke for a given change in the level of that IP used in the first analysis. Where studies reported different ORs for different ranges of IP level, we chose the OR reported for the range closest to that applying to the control group (i.e. healthy population). The ORs between studies were pooled using a generic inverse variance procedure in which the logarithms of the ORs are weighted according to variance in a random-effects model. Since the ORs reported between these studies usually refer to different amounts of IP change, we first scaled the OR values for each study in proportion to the pooled change in IP level for the genotype comparison of interest (from the first analysis) assuming a log-linear relationship [11]. For the relationship between homocysteine levels and ischemic stroke we used a summary OR from a previous meta-analysis [24].

## Supporting Information

Figures S1 Supplementary Figures 1–10: Forest plots of genetic polymorphisms tested in more than 1000 pooled cases but showing no association with ischemic stroke.
(0.08 MB PDF)

References S1 Supplementary References
(0.29 MB PDF)
