# Supplementary material for: Causal Relationship of Susceptibility Genes to Ischemic Stroke: Comparison to Ischemic Heart Disease and Biochemical Determinants
Source: PLoS One. 2010 Feb 9;5(2):e9136. doi: 10.1371/journal.pone.0009136 (PMC2817726; doi:10.1371/journal.pone.0009136)

Supplementary Fig. 1

Phosphodiesterase 4D
(PDE 4D SNP45*)
AA vs G+

Study or Subgroup	Stroke Events	Stroke Total	Control Events	Control Total	Weight	Odds Ratio M-H, Random, 95% CI
Zee RY 2006	3	259	8	259	5.1%	0.37 [0.10, 1.40]
Gretarsdottir S 2003	19	723	25	492	12.9%	0.50 [0.27, 0.93]
Meschia JF 2005	25	376	29	262	13.8%	0.57 [0.33, 1.00]
Kostulas K 2007	8	373	18	571	9.5%	0.67 [0.29, 1.56]
Bevan S 2005	21	734	29	928	13.6%	0.91 [0.52, 1.61]
Lohmussr E 2005	14	595	17	734	11.2%	1.02 [0.50, 2.08]
Kuhlenbaumer G 2006	31	1157	29	1563	14.6%	1.46 [0.87, 2.43]
Nilsson-Ardnor 2005	6	220	6	442	6.4%	2.04 [0.65, 6.39]
Fidani L 2007	4	97	2	102	3.4%	2.15 [0.38, 12.02]
Van Rijn MJ 2005	5	89	5	191	5.6%	2.21 [0.62, 7.85]
Staton JM 2006	6	151	2	164	3.8%	3.35 [0.67, 16.87]
**Total (95% CI)**		**4774**		**5708**	**100.0%**	**0.95 [0.67, 1.35]**
Total events	142		170			

Heterogeneity: $Tau^2 = 0.15$; $Chi^2 = 19.11$, df = 10 (P = 0.04); $I^2 = 48\%$
Test for overall effect: Z = 0.27 (P = 0.79)

Odds Ratio
M-H, Random, 95% CI

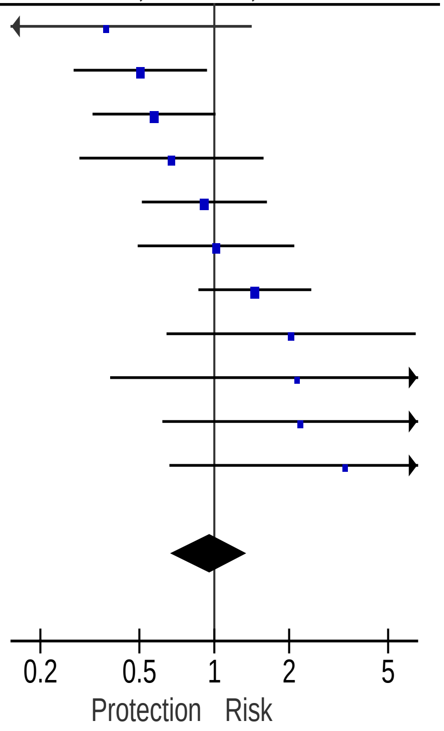

* Negative results were similarly found for PDE 4D polymorphisms SNP 26, 56, 83, 87, 89 (i.e. those for which data on >1000 pooled cases exist); using dominant or recessive genetic models for all.

Supplementary Fig. 2

Apolipoprotein E
(Apo E4)
E4 vs not E4 allele*

Study or Subgroup	Stroke Events	Stroke Total	Control Events	Control Total	Weight	Odds Ratio M-H, Random, 95% CI
Duzenli S 2004	2	62	22	126	2.2%	0.16 [0.04, 0.69]
Margaglione M 1998	13	108	30	100	4.9%	0.32 [0.16, 0.66]
Cerrato P 2005	32	302	42	228	6.2%	0.52 [0.32, 0.86]
MacLeod MJ 2001	66	266	72	225	6.7%	0.70 [0.47, 1.04]
Slowik A 2003	15	71	8	30	3.7%	0.74 [0.27, 1.98]
Tasdemir N 2008	14	100	5	30	3.2%	0.81 [0.27, 2.48]
Basun H 1996	31	132	207	794	6.5%	0.87 [0.56, 1.34]
Catto AJ 2000	133	515	82	289	7.1%	0.88 [0.64, 1.21]
McIlroy SP 2002	11	64	13	71	4.1%	0.93 [0.38, 2.24]
Abboud S 2008	60	237	82	326	6.8%	1.01 [0.69, 1.48]
Karttunen V 2002	14	44	32	104	4.7%	1.05 [0.49, 2.24]
Frikke-Schmidt R 2001	94	286	2717	8938	7.4%	1.12 [0.87, 1.44]
Kessler C 1997	55	220	49	223	6.5%	1.18 [0.76, 1.84]
Couderc R 1993	11	69	9	68	3.8%	1.24 [0.48, 3.22]
Traykov L 2007	15	50	30	122	4.8%	1.31 [0.63, 2.73]
Hachinski V 1996	28	89	20	89	5.2%	1.58 [0.81, 3.09]
Pezzini A 2005	40	163	22	158	5.7%	2.01 [1.13, 3.57]
Topic E 2001	9	56	9	124	3.7%	2.45 [0.91, 6.55]
Saidi S 2007	132	216	94	282	6.9%	3.14 [2.17, 4.55]
**Total (95% CI)**		**3050**		**12327**	**100.0%**	**1.02 [0.79, 1.32]**
Total events	775		3545			

Heterogeneity: $Tau^2 = 0.21$; $Chi^2 = 75.00$, df = 18 ($P < 0.00001$); $I^2 = 76\%$
Test for overall effect: Z = 0.15 (P = 0.88)

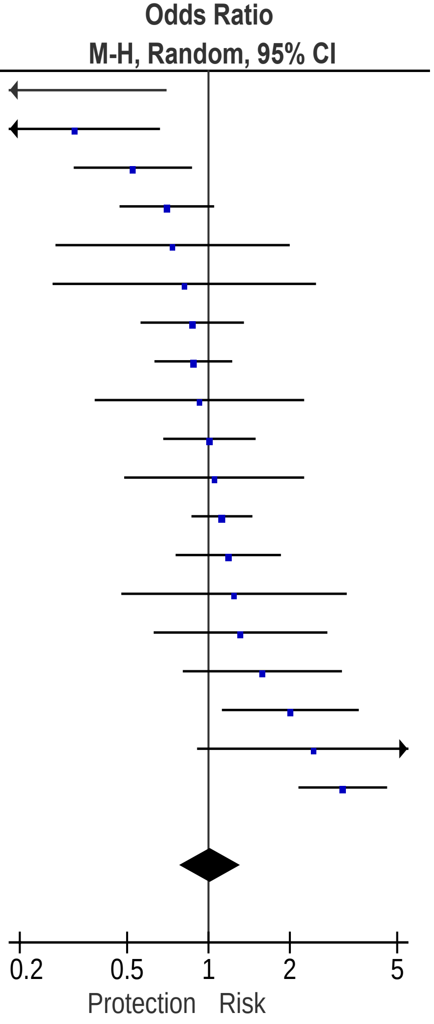

* Negative results were similarly found for other Apo E genetic models

Supplementary Fig. 3

Factor XIII
(Val34Leu)
LeuLeu vs Val+

Study or Subgroup	Stroke Events	Stroke Total	Control Events	Control Total	Weight	Odds Ratio M-H, Random, 95% CI
Corral J 2000	2	104	4	104	3.5%	0.49 [0.09, 2.74]
Gemmati D 2001	10	240	15	240	11.1%	0.65 [0.29, 1.48]
Elbaz A 2000	27	456	34	456	18.0%	0.78 [0.46, 1.32]
Endler G 2003	54	718	32	369	20.1%	0.86 [0.54, 1.35]
Catto AJ 1998	46	612	25	436	18.6%	1.34 [0.81, 2.21]
Rubattu S 2005	15	294	10	286	11.1%	1.48 [0.66, 3.36]
Slowik A 2005	9	66	10	135	9.0%	1.97 [0.76, 5.12]
Reiner AP 2002	6	36	20	345	8.6%	3.25 [1.21, 8.71]
**Total (95% CI)**		**2526**		**2371**	**100.0%**	**1.12 [0.80, 1.57]**
Total events	169		150			

Heterogeneity: $Tau^2 = 0.10$; $Chi^2 = 12.34$, df = 7 (P = 0.09); $I^2 = 43\%$
Test for overall effect: Z = 0.65 (P = 0.51)

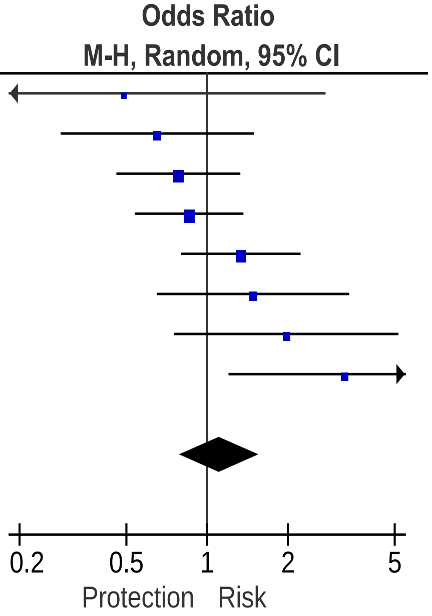

Supplementary Fig. 4

Angiotensinogen
(M235T)
TT vs M+

	Stroke		Control			Odds Ratio	Odds Ratio
**Study or Subgroup**	**Events**	**Total**	**Events**	**Total**	**Weight**	**M-H, Random, 95% CI**	**M-H, Random, 95% CI**
Barley J 1995	12	100	8	45	2.2%	0.63 [0.24, 1.67]	
Sethi A 2001	139	923	1310	7975	57.8%	0.90 [0.75, 1.09]	
Brenner D 2005	74	459	78	459	17.2%	0.94 [0.66, 1.33]	
Gormley K 2007	40	284	86	596	12.7%	0.97 [0.65, 1.46]	
Bis JC 2003	30	116	153	717	10.2%	1.29 [0.82, 2.02]	
**Total (95% CI)**		**1882**		**9792**	**100.0%**	**0.94 [0.82, 1.09]**	
Total events	295		1635				

Heterogeneity: $Tau^2 = 0.00$; $Chi^2 = 2.69$, df = 4 (P = 0.61); $I^2 = 0\%$
Test for overall effect: Z = 0.79 (P = 0.43)

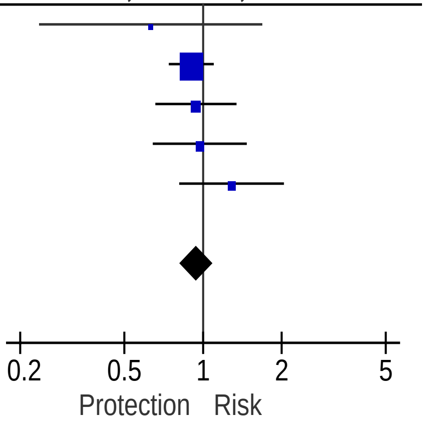

* Negative results were similarly found for Angiotensinogen T147M polymorphism. MM vs T+: OR=0.85 (95% CIs: 0.50- 1.43).

Supplementary Fig. 5

Glycoprotein 1b-α
(Kozak sequence TT)
TT vs C+

	Stroke		Control			Odds Ratio
**Study or Subgroup**	**Events**	**Total**	**Events**	**Total**	**Weight**	**M-H, Random, 95% CI**
Corral J 2000	82	104	87	104	23.8%	0.73 [0.36, 1.47]
Frank MB 2001	31	41	293	384	23.2%	0.96 [0.45, 2.04]
Hsieh K 2004	1049	1399	763	1066	28.7%	1.19 [0.99, 1.42]
Baker RI 2001	135	205	14	61	24.3%	6.47 [3.34, 12.56]
**Total (95% CI)**		**1749**		**1615**	**100.0%**	**1.52 [0.68, 3.38]**
Total events	1297		1157			

Heterogeneity: $Tau^2 = 0.57$; $Chi^2 = 26.68$, df = 3 ($P < 0.00001$); $I^2 = 89\%$
Test for overall effect: Z = 1.03 (P = 0.30)

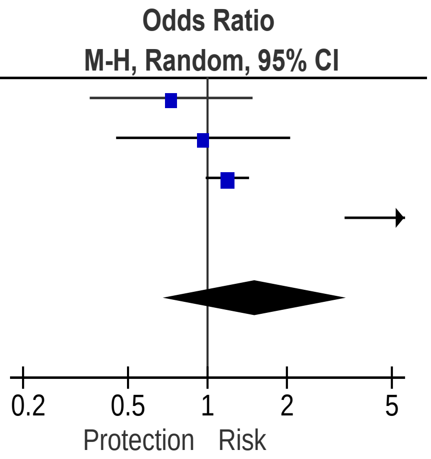

Supplementary Fig. 6

eNOS
(G894T)
AspAsp vs Glu+

	Stroke		Control			Odds Ratio	Odds Ratio
**Study or Subgroup**	**Events**	**Total**	**Events**	**Total**	**Weight**	**M-H, Random, 95% CI**	**M-H, Random, 95% CI**
Guldiken B 2009	5	146	6	133	3.5%	0.75 [0.22, 2.52]	
Markus HS 1998	47	361	36	236	21.2%	0.83 [0.52, 1.33]	
Elbaz A 2000	61	460	65	460	31.1%	0.93 [0.64, 1.35]	
MaCleod MJ 1999	31	265	36	393	18.4%	1.31 [0.79, 2.18]	
Hassan A 2001	42	295	62	595	25.8%	1.43 [0.94, 2.17]	
**Total (95% CI)**		**1527**		**1817**	**100.0%**	**1.07 [0.85, 1.35]**	
Total events	186		205				

Heterogeneity: $Tau^2 = 0.01$; $Chi^2 = 4.42$, df = 4 (P = 0.35); $I^2 = 10\%$
Test for overall effect: Z = 0.60 (P = 0.55)

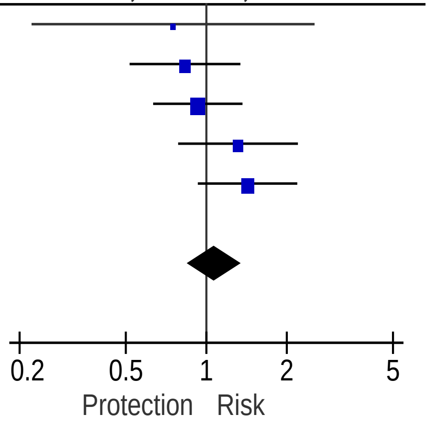

Supplementary Fig. 7

IL-6
(G174C)
GG vs C+

Study or Subgroup	Stroke Events	Stroke Total	Control Events	Control Total	Weight	Odds Ratio M-H, Random, 95% CI
Revilla M 2002	27	82	37	82	12.1%	0.60 [0.32, 1.12]
Chamorro A 2005	104	273	46	105	14.3%	0.79 [0.50, 1.25]
Lalouschek W 2006	143	404	156	416	16.2%	0.91 [0.69, 1.21]
Balding J 2004	33	105	123	389	14.2%	0.99 [0.62, 1.58]
Greisenegger S 2003	81	214	76	214	15.1%	1.11 [0.75, 1.64]
Flex A 2004	100	237	56	224	15.0%	2.19 [1.47, 3.26]
Pola R 2003	56	119	28	133	13.1%	3.33 [1.92, 5.78]
**Total (95% CI)**		**1434**		**1563**	**100.0%**	**1.20 [0.81, 1.76]**
Total events	544		522			

Heterogeneity: $Tau^2 = 0.22$; $Chi^2 = 34.11$, df = 6 (P < 0.00001); $I^2 = 82\%$
Test for overall effect: Z = 0.90 (P = 0.37)

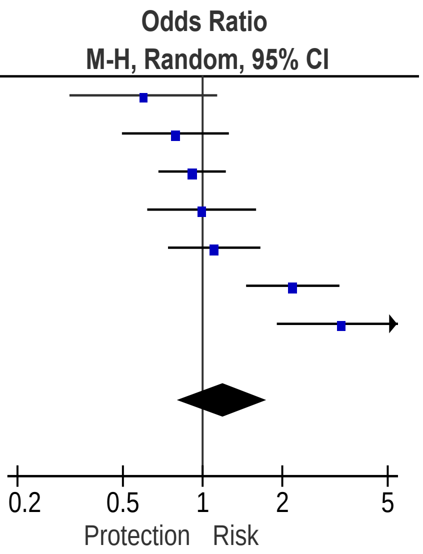

Supplementary Fig. 8

HFE
(C282Y*)
Y+ vs CC

Study or Subgroup	Stroke Events	Stroke Total	Control Events	Control Total	Weight	Odds Ratio M-H, Random, 95% CI
Ekblom K 2005	19	222	49	445	13.4%	0.76 [0.43, 1.32]
Ellervik C 2007	40	393	936	8577	37.0%	0.93 [0.66, 1.29]
Hetet G 2001	46	441	44	441	21.7%	1.05 [0.68, 1.63]
Hruskovicoca H 2005	7	96	8	115	3.7%	1.05 [0.37, 3.01]
Njajou OT 2002	28	202	383	2730	24.2%	0.99 [0.65, 1.49]
**Total (95% CI)**		**1354**		**12308**	**100.0%**	**0.94 [0.77, 1.16]**
Total events	140		1420			

Heterogeneity: Tau² = 0.00; Chi² = 0.94, df = 4 (P = 0.92); I² = 0%
Test for overall effect: Z = 0.55 (P = 0.58)

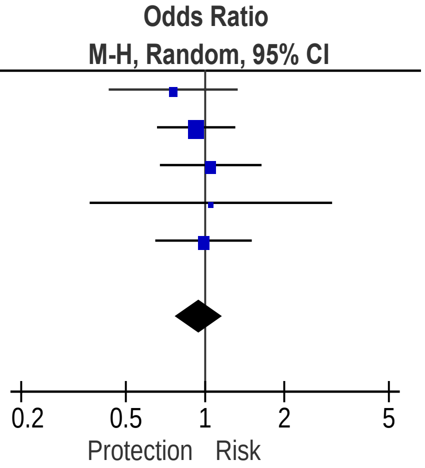

* Negative results were similarly found for HFE polymorphism H63D: D+ VS HH: OR=1.08 (95% CIs: 0.94 - 1.25); 1344 cases; 12296 controls.

Supplementary Fig. 9

Angiotensin Receptor
(A1166C)
C+ vs AA

Study or Subgroup	Stroke Events	Stroke Total	Control Events	Control Total	Weight	Odds Ratio M-H, Random, 95% CI
Mollsten A 2008	91	220	269	549	25.1%	0.73 [0.54, 1.01]
Szolnoki Z 2006	158	308	144	272	24.9%	0.94 [0.68, 1.30]
Rubattu S 2004	125	215	112	236	24.2%	1.54 [1.06, 2.23]
Brenner D 2005	233	459	149	459	25.8%	2.14 [1.64, 2.80]
**Total (95% CI)**		**1202**		**1516**	**100.0%**	**1.23 [0.74, 2.04]**
Total events	607		674			

Heterogeneity: $Tau^2 = 0.24$; $Chi^2 = 30.61$, df = 3 ($P < 0.00001$); $I^2 = 90\%$
Test for overall effect: Z = 0.80 (P = 0.42)

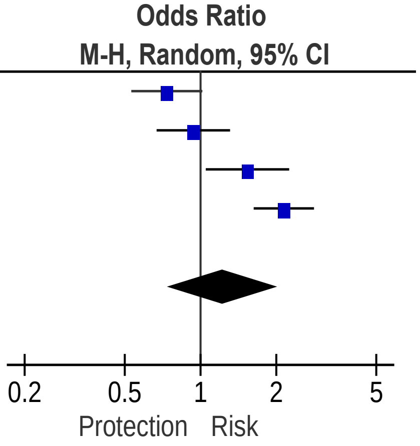

Supplementary Fig. 10

Factor VII
(R353Q)
Q+ vs RR

	Stroke		Control			Odds Ratio	Odds Ratio
**Study or Subgroup**	**Events**	**Total**	**Events**	**Total**	**Weight**	**M-H, Random, 95% CI**	**M-H, Random, 95% CI**
Zee RY 2004	63	319	538	2092	24.2%	0.71 [0.53, 0.95]	
Heywood DM 1997	64	299	44	178	20.7%	0.83 [0.53, 1.29]	
Corral J 1998	31	104	23	104	16.3%	1.50 [0.80, 2.79]	
Rubattu S 2005	112	294	83	286	22.9%	1.51 [1.06, 2.13]	
Petrovic D 2003	31	96	22	105	16.0%	1.80 [0.95, 3.40]	
**Total (95% CI)**		**1112**		**2765**	**100.0%**	**1.14 [0.77, 1.69]**	
Total events	301		710				

Heterogeneity: Tau² = 0.14; Chi² = 16.03, df = 4 (P = 0.003); I² = 75%
Test for overall effect: Z = 0.66 (P = 0.51)

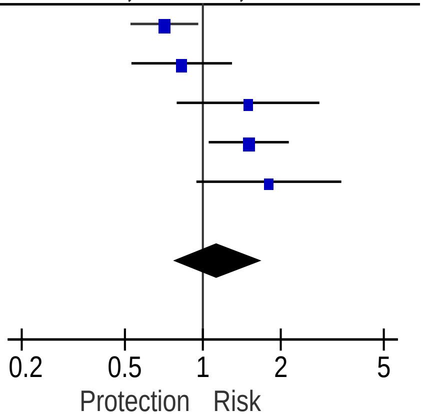

Supplement: Figures S1 — Supplementary Figures 1– 10: Forest plots of genetic polymorphisms tested in more than 1000 pooled cases but showing no association with ischemic stroke. (0.08 MB PDF) [file pone.0009136.s001.pdf]
