# Supplementary material for: Causal Relationship of Susceptibility Genes to Ischemic Stroke: Comparison to Ischemic Heart Disease and Biochemical Determinants
Source: PLoS One. 2010 Feb 9;5(2):e9136. doi: 10.1371/journal.pone.0009136 (PMC2817726; doi:10.1371/journal.pone.0009136)
Supplement: References S1 — Supplementary References (0.29 MB PDF) [file pone.0009136.s002.pdf]

Supplementary References

Stroke Meta-Analyses References (Categorised by Gene)

FACTOR V LEIDEN

Factor V Leiden Gene - Stroke

Albucher JF, Guiraud-Chaumeil B, Chollet F, Cadroy Y, Sie P. Frequency of resistance to activated protein C due to factor V mutation in young patients with ischemic stroke. Stroke.1996;27:766-767

Aznar J, Mira Y, Vaya A, et al. Factor V Leiden and prothrombin G20210A mutations in young adults with cryptogenic ischemic stroke. Thromb Haemost. 2004;91(5):1031-4

Bentolila S, Ripoll L, Drouet L, Mazoyer E, Woimant F. Thrombophilia due to 20210 G-->A prothrombin polymorphism and cerebral ischemia in the young. Stroke. 1997;28:1846-1847

Buyru N, Altinisik J, Somay G, Ulutin T. Factor V Leiden mutation in cerebrovascular disease. Clin Appl Thromb Hemost. 2005;11(3):339-42

Catto A, Carter AM, Ireland H et al. Factor V Leiden gene mutation and thrombin generation in relation to the development of acute stroke. Arterioscler Thromb Vasc Biol. 1995;15:783-785

Chimowitz M, Mansbach H, Schmaier A, Nichols W, Ginsburg D. Factor V mutation and cryptogenic stroke in the young (abstract). Stroke. 1996;27:188

DeStefano V, Chiusolo P, Paciaroni K, et al. Prothrombin G20210A mutant genotype is a risk factor for cerebrovascular ischaemic disease in young patients. Blood. 1998;91:3562-3565

Eterović D, Titlić M, Culić V, Zadro R, Primorac D. Lower contribution of factor V Leiden or G202104 mutations to ischemic stroke in patients with clinical risk factors: pair-matched case-control study. Clin Appl Thromb Hemost. 2007;13(2):188-93.

Halbmayer WM, Haushofer A, Angerer V, Finsterer J, Fischer M. APC resistance and factor V leiden mutation in patients with ischaemic cerebral events. Blood Coagul Fibrinolysis. 1997;8:361-364

Hankey GJ, Eikelboom JW, van Bockxmeer FM, Lofthouse E, Staples N, Baker RI. Inherited thrombophilia in ischaemic stroke and its pathogenic subtypes. Stroke. 2001;32:1793-1799

Iniesta JA, Corral J, Gonzalez-Conejero R, Rivera J, Vicente V. Prothrombotic genetic risk factors in patients with coexisting migraine and ischemic cerebrovascular disease. Headache. 1999;39:486-489.

Juul K, Tybjaerg-Hansen A, Steffensen R, Kofoed S, Jensen G, Nordestgaard BG. Factor V Leiden: The Copenhagen City Heart Study and 2 meta-analyses. Blood. 2002;100:3-10

Kontula K, Ylikorkala A, Miettinen H, et al. Arg506Gln factor V mutation (factor V Leiden) in patients with ischaemic cerebrovascular disease and survivors of myocardial infarction. Thromb Haemost. 1995;73(4):558-60

Lalouschek W, Aull S, Series W, Zeiler K, Mannhalter C. The prothrombin G20210A mutation and factor V Leiden mutation in patients with cerebrovascular disease. Blood. 1998 ;92(2):704-5.

Lalouschek W, Schillinger M, Hsieh K, et al. Matched case-control study on factor V Leiden and the prothrombin G20210A mutation in patients with ischemic stroke/transient ischemic attack up to the age of 60 years. Stroke. 2005;36(7):1405-9

Landi G, Cella E, Martinelli I, Tagliabue L, Mannucci PM, Zerbi D. Arg506Gln factor V mutation and cerebral ischemia in the young. Stroke. 1996;27:1697-1698

Lopaciuk S, Bykowska K, Kwiecinski H, et al. Factor V Leiden, prothrombin gene G20210A variant, and methylenetetrahydrofolate reductase C677T genotype in young adults with ischemic stroke. Clin Appl Thromb Hemost. 2001;7:346-350.

Madonna P, de Stefano V, Coppola A, et al. Hyperhomocysteinemia and other inherited prothrombotic conditions in young adults with a history of ischemic stroke. Stroke. 2002;33:51-56.

Margaglione M, D'Andrea G, Giuliani N, et al. Inherited prothrombotic conditions and premature ischemic stroke: sex difference in the association with factor V Leiden. Arterioscler Thromb Vasc Biol. 1999;19:1751-1756.

Markus HS, Zhang Y, Jeffery S. Screening for the factor-V Arg 506 Gln mutation in patients with TIA and stroke. Cerebrovasc Dis. 1996;6:360-362.

Martinelli I, Franchi F, Akwan S, Bettini P, Merati G, Mannucci PM. The transition G to A at position 20210 in the 3'-untranslated region of the prothrombin gene is not associated with cerebral ischemia [letter]. Blood. 1997;90:3806.

Meseguer E, Llamas P, Fernandez de Velasco J, et al. Prothrombotic factors in stroke (Spanish). Neurologia. 2004;19(3):99-105

Nabavi DG, Junker R, Wolff E, et al. Prevalence of factor V Leiden mutation in young adults with cerebral ischaemia: a case-control study on 225 patients. J Neurol. 1998;245:653-658.

Petrovic D, Milanez T, Kobal J, Bregar D, Potisk KP, Peterlin B. Prothrombotic gene polymorphisms and atherothrombotic cerebral infarction. Acta Neurol Scand. 2003;108(2):109-13

Pezzini A, Del Zotto E, Magoni M, et al. Inherited thrombophilic disorders in young adults with ischemic stroke and patent foramen ovale. Stroke. 2003;34:28-33.

Pezzini A, Grassi M, Del Zotto E, et al. Cumulative effect of predisposing genotypes and their interaction with modifiable factors on the risk of ischemic stroke in young adults. Stroke. 2005;36(3):533-9

Press RD, Liu XY, Beamer N, Coull BM. Ischemic stroke in the elderly: role of the common factor V mutation causing resistance to activated protein C. Stroke. 1996;27:44-48.

Ridker PM, Hennekens CH, Lindpaintner K, Stampfer MJ, Eisenberg PR, Miletich JP. Mutation in the gene coding for coagulation factor V and the risk of myocardial infarction, stroke, and venous thrombosis in apparently healthy men. N Engl J Med. 1995;332:912-917.

Rubattu S, Di Angelantonio E, Nitsch D, et al. Polymorphisms in prothrombotic genes and their impact on ischemic stroke in a Sardinian population. Thromb Haemost. 2005;93(6):1095-100

Sanchez J, Roman J, de la Torre MJ, Velasco F, Torres A. Low prevalence of the factor V Leiden among patients with ischemic stroke. Haemostasis. 1997;27:9-15

Szolnoki Z, Somogyvari F, Kondacs A, Szabo M, Fodor L, Bene J, Melegh B. Evaluation of the modifying effects of unfavourable genotypes on classical clinical risk factors for ischaemic stroke. J Neurol Neurosurg Psychiatry. 2003;74(12):1615-20.

van der Bom JG, Bots ML, Haverkate F, et al. Reduced response to activated protein C is associated with increased risk for cerebrovascular disease. Ann Intern Med. 1996;125:265-269.

Voetsch B, Damasceno BP, Camargo EC, et al. Inherited thrombophilia as a risk factor for the development of ischemic stroke in young adults. Thromb Haemost. 2000;83:229-233.

Zunker P, Hohenstein C, Plendl HJ, et al. Activated protein C resistance and acute ischaemic stroke: relation to stroke causation and age. J Neurol. 2001;248:701-704.

Factor V Leiden Gene - Activated Protein C Resistance

De Bel AV, Van der Cruyssen GA, Devreese KM. A 2-year retrospective analysis of laboratory testing for activated protein C resistance with a factor V-corrected activated partial thromboplastin time-based method. Blood Coagul Fibrinolysis. 2006;17(2):155-60.

Lowe GD, Rumley A, Woodward M, Reid E, Rumley J. Activated protein C resistance and the FV:R506Q mutation in a random population sample--associations with cardiovascular risk factors and coagulation variables. Thromb Haemost. 1999;81(6):918-24.

Wilmer M, Stocker C, Bühler B, Conell B, Calatzis A. Improved distinction of factor V wild-type and factor V Leiden using a novel prothrombin-based activated protein C resistance assay. Am J Clin Pathol. 2004;122(6):836-42.

Activated Protein C Resistance – Stroke

Smith A, Patterson C, Yarnell J, Rumley A, Ben-Shlomo Y, Lowe G. Which

hemostatic markers add to the predictive value of conventional risk factors for coronary heart disease and ischemic stroke? The Caerphilly Study. Circulation. 2005;112(20):3080-7.

van der Bom JG, Bots ML, Haverkate F, Slagboom PE, Meijer P, de Jong PT, Hofman A, Grobbee DE, Kluft C. Reduced response to activated protein C is associated with increased risk for cerebrovascular disease. Ann Intern Med. 1996;125(4):265-9.

ACE – ID

ACE Gene – Stroke

Agerholm-Larsen B, Tybjaerg-Hansen A, Frikke-Schmidt R, Gronholdt ML, Jensen G, Nordestgaard BG. ACE gene polymorphism as a risk factor for ischemic cerebrovascular disease. Ann Intern Med. 1997;127:346-355.

Brenner D, Labreuche J, Poirier O, Cambien F, Amarenco P; GENIC Investigators. Renin-angiotensin-aldosterone system in brain infarction and vascular death. Ann Neurol. 2005 Jul;58(1):131-8

Catto A, Carter AM, Barrett JH, et al. Angiotensin-converting enzyme insertion/deletion polymorphism and cerebrovascular disease. Stroke. 1996;27:435-440.

Dikmen M, Günes HV, Degirmenci I, Ozdemir G, Basaran A**.** Are the angiotensin-converting enzyme gene and activity risk factors for stroke? Arq Neuropsiquiatr. 2006 Jun;64(2A):211-6.

Gormley K, Bevan S, Markus HS. Polymorphisms in genes of the renin-angiotensin system and cerebral small vessel disease. Cerebrovasc Dis. 2007;23(2-3):148-55.

Karagiannis A, Balaska K, Tziomalos K, Tokalaki-Nikolaidou L, Papayeoryiou A, Zamboulis C. Lack of an association between angiotensin-converting enzyme gene insertion/deletion polymorphism and ischaemic stroke. Eur Neurol. 2004;51(3):148-52.

Kostulas K, Huang WX, Crisby M, et al. An angiotensin-converting enzyme gene polymorphism suggests a genetic distinction between ischaemic stroke and carotid stenosis. Eur J Clin Invest. 1999;29:478-483.

Margaglione M, Celentano E, Grandone E, et al. Deletion polymorphism in the angiotensin-converting enzyme gene in patients with a history of ischemic stroke. Arterioscler Thromb Vasc Biol. 1996;16:304-309.

Markus HS, Barley J, Lunt R, et al. Angiotensin-converting enzyme gene deletion polymorphism: a new risk factor for lacunar stroke but not carotid atheroma. Stroke. 1995;26:1329-1333.

Möllsten A, Stegmayr B, Wiklund PG. Genetic polymorphisms in the renin-angiotensin system confer increased risk of stroke independently of blood pressure: a nested case-control study. J Hypertens. 2008;26(7):1367-72.

Peterlin B, Petrovic D, Zorc M, Keber I. Deletion/insertion polymorphism in the angiotension-converting enzyme gene as a risk factor in the Slovenian patients with coronary heart disease. Pflugers Arch. 2000;439(suppl):R40-R41.

Pera J, Slowik A, Dziedzic T, Wloch D, Szczudlik A. ACE I/D polymorphism in different etiologies of ischemic stroke. Acta Neurol Scand. 2006;114(5):320-2.

Pfohl M, Fetter M, Koch M, Barth CM, Rudiger W, Haring HU. Association between angiotensin I–converting enzyme genotypes, extracranial artery stenosis, and stroke. Atherosclerosis. 1998;140:161-166.

Sharma P, Carter ND, Barley J, Brown MM. Molecular approach to assessing the genetic risk of cerebral infarction: deletion polymorphism in the gene encoding angiotensin 1–converting enzyme. J Hum Hypertens. 1994;8:645-648.

Slowik A, Turaj W, Dziedzic T, et al. DD genotype of ACE gene is a risk factor for intracerebral hemorrhage. Neurology. 2004;63(2):359-61

Szolnoki Z, Somogyvari F, Kondacs A, Szabo M, Fodor L, Bene J, Melegh B. Evaluation of the modifying effects of unfavourable genotypes on classical clinical risk factors for ischaemic stroke. J Neurol Neurosurg Psychiatry. 2003;74(12):1615-20.

Tuncer N, Tuglular S, Kilic G, Sazci A, Us O, Kara I. Evaluation of the angiotensin-converting enzyme insertion/deletion polymorphism and the risk of ischaemic stroke. J Clin Neurosci. 2006;13(2):224-227

Ueda S, Weir CJ, Inglis GC, Murray GD, Muir KW, Lees KR. Lack of association between angiotensin converting enzyme gene insertion/deletion polymorphism and stroke. J Hypertens. 1995;13:1597-1601.

Zee RY, Ridker PM, Stampfer MJ, Hennekens CH, Lindpaintner K. Prospective evaluation of the angiotensin-converting enzyme insertion/deletion polymorphism and the risk of stroke. Circulation. 1999;99:340-343.

ACE Gene – ACE Activity

Agerholm-Larsen B, Tybjaerg-Hansen A, Schnohr P, Nordestgaard BG. ACE gene polymorphism explains 30-40% of variability in serum ACE activity in both women and men in the population at large: the Copenhagen City Heart Study. Atherosclerosis. 1999;147(2):425-7.

Arbustini E, Grasso M, Leo G, Tinelli C, Fasani R, Diegoli M, Banchieri N, Cipriani A, Gorrini M, Semenzato G, Luisetti M. Polymorphism of angiotensin-converting enzyme gene in sarcoidosis. Am J Respir Crit Care Med. 1996;153(2):851-4.

Busjahn A, Knoblauch H, Knoblauch M, Bohlender J, Menz M, Faulhaber HD, Becker A, Schuster H, Luft FC. Angiotensin-converting enzyme and angiotensinogen gene polymorphisms, plasma levels, cardiac dimensions. A twin study. Hypertension. 1997;29(1 Pt 2):165-70.

Cambien F, Costerousse O, Tiret L, Poirier O, Lecerf L, Gonzales MF, Evans A, Arveiler D, Cambou JP, Luc G, et al. Plasma level and gene polymorphism of angiotensin-converting enzyme in relation to myocardial infarction. Circulation. 1994;90(2):669-76.

Catto A, Carter AM, Barrett JH, Stickland M, Bamford J, Davies JA, Grant PJ. Angiotensin-converting enzyme insertion/deletion polymorphism and cerebrovascular disease. Stroke. 1996;27(3):435-40.

Cídl K, Strelcová L, Znojil V, Váchi J. Angiotensin I-converting enzyme (ACE) polymorphism and ABO blood groups as factors codetermining plasma ACE activity. Exp Hematol. 1996;24(7):790-4.

Danilov S, Savoie F, Lenoir B, Jeunemaitre X, Azizi M, Tarnow L, Alhenc-Gelas F. Development of enzyme-linked immunoassays for human angiotensin I converting enzyme suitable for large-scale studies. J Hypertens. 1996;14(6):719-27.

Day SH, Gohlke P, Dhamrait SS, Williams AG. No correlation between circulating ACE activity and VO2max or mechanical efficiency in women. Eur J Appl Physiol. 2007;99(1):11-8.

Dessi-Fulgheri P, Catalini R, Sarzani R, Sturbini S, Siragusa N, Guazzarotti F, Offidani M, Tamburrini P, Zingaretti O, Rappelli A. Angiotensin converting enzyme gene

polymorphism and carotid atherosclerosis in a low-risk population. J Hypertens. 1995;13(12 Pt 2):1593-6.

Faure-Delanef L, Baudin B, Bénéteau-Burnat B, Beaudoin JC, Giboudeau J, Cohen D. Plasma concentration, kinetic constants, and gene polymorphism of angiotensin I-converting enzyme in centenarians. Clin Chem. 1998;44(10):2083-7.

Foy CA, McCormack LJ, Knowler WC, Barrett JH, Catto A, Grant PJ. The angiotensin-I converting enzyme (ACE) gene I/D polymorphism and ACE levels in Pima Indians. J Med Genet. 1996;33(4):336-7.

Freire MB, van Dijk DJ, Erman A, Boner G, Warram JH, Krolewski AS. DNA polymorphisms in the ACE gene, serum ACE activity and the risk of nephropathy in insulin-dependent diabetes mellitus. Nephrol Dial Transplant. 1998;13(10):2553-8.

Frossard PM, Lestringant GG, Obineche EN, Hill SH. The angiotensin-converting enzyme (ACE) gene insertion/deletion dimorphism tracks with higher serum ace activities in both younger and older subjects. Ann Saudi Med. 1998;18(5):389-92.

Gardemann A, Weiss T, Schwartz O, Eberbach A, Katz N, Hehrlein FW, Tillmanns H, Waas W, Haberbosch W. Gene polymorphism but not catalytic activity of angiotensin I-converting enzyme is associated with coronary artery disease and myocardial infarction in low-risk patients. Circulation. 1995;92(10):2796-9.

Harrap SB, Davidson HR, Connor JM, Soubrier F, Corvol P, Fraser R, Foy CJ, Watt GC. The angiotensin I converting enzyme gene and predisposition to high blood pressure. Hypertension. 1993;21(4):455-60.

Huang S, Chen XH, Payne JR, Pennell DJ, Gohlke P, Smith MJ, Day IN, Montgomery HE, Gaunt TR. Haplotype of growth hormone and angiotensin I-converting enzyme

genes, serum angiotensin I-converting enzyme and ventricular growth: pathway inference in pharmacogenetics. Pharmacogenet Genomics. 2007;17(4):291-4.

Jalil JE, Ocaranza MP, Oliveri C, Córdova S, Godoy I, Chamorro G, Braun S, Fardella C, Michel JB, Lavandero S. Neutral endopeptidase and angiotensin I converting enzyme insertion/deletion gene polymorphism in humans. J Hum Hypertens. 2004;18(2):119-25.

Markus HS, Barley J, Lunt R, Bland JM, Jeffery S, Carter ND, Brown MM. Angiotensin-converting enzyme gene deletion polymorphism. A new risk factor for lacunar stroke but not carotid atheroma. Stroke. 1995;26(8):1329-33.

McKenzie CA, Julier C, Forrester T, McFarlane-Anderson N, Keavney B, Lathrop GM, Ratcliffe PJ, Farrall M. Segregation and linkage analysis of serum angiotensin I-converting enzyme levels: evidence for two quantitative-trait loci. Am J Hum Genet. 1995;57(6):1426-35.

Morris BJ, Zee RY, Schrader AP. Different frequencies of angiotensin-converting enzyme genotypes in older hypertensive individuals. J Clin Invest. 1994;94(3):1085-9.

Pereira AC, Morandini Filho AA, Heimann AS, Rabak ET, Vieira AP, Mota GF, Krieger JE. Serum angiotensin converting enzyme activity association with the I/D polymorphism in an ethnically admixtured population. Clin Chim Acta. 2005;360(1-2):201-4.

Rice GI, Jones AL, Grant PJ, Carter AM, Turner AJ, Hooper NM. Circulating activities of angiotensin-converting enzyme, its homolog, angiotensin-converting enzyme 2, and neprilysin in a family study. Hypertension. 2006;48(5):914-20.

Rigat B, Hubert C, Alhenc-Gelas F, Cambien F, Corvol P, Soubrier F. An insertion/deletion polymorphism in the angiotensin I-converting enzyme gene accounting for half the variance of serum enzyme levels. J Clin Invest. 1990;86(4):1343-6.

Sanders J, Harris J, Cooper J, Gohlke P, Humphries SE, Montgomery H, Woods DR. Lack of change in serum angiotensin-converting enzyme activity during the menstrual cycle. J Renin Angiotensin Aldosterone Syst. 2006;7(4):231-5.

Tiret L, Rigat B, Visvikis S, Breda C, Corvol P, Cambien F, Soubrier F. Evidence, from combined segregation and linkage analysis, that a variant of the angiotensin I-converting enzyme (ACE) gene controls plasma ACE levels. Am J Hum Genet. 1992;51(1):197-205.

Winkelmann BR, Nauck M, Klein B, Russ AP, Böhm BO, Siekmeier R, Ihnken K, Verho M, Gross W, März W. Deletion polymorphism of the angiotensin I-converting enzyme gene is associated with increased plasma angiotensin-converting enzyme activity but not with increased risk for myocardial infarction and coronary artery disease. Ann Intern Med. 1996;125(1):19-25.

Woods D, Sanders J, Jones A, Hawe E, Gohlke P, Humphries SE, Payne J, Montgomery H. The serum angiotensin-converting enzyme and angiotensin II response to altered posture and acute exercise, and the influence of ACE genotype. Eur J Appl Physiol. 2004;91(2-3):342-8.

<u>ACE Gene – Stroke</u>

Brenner D, Labreuche J, Poirier O, Cambien F, Amarenco P; GENIC Investigators. Renin-angiotensin-aldosterone system in brain infarction and vascular death. Ann Neurol. 2005;58(1):131-8.

<u>MTHFR</u>

<u>MTHFR Gene – Stroke</u>

De Stefano V, Chiusolo P, Paciaroni K, et al. Prothrombin G20210A mutant genotype is a risk factor for cerebrovascular ischemic disease in young patients. Blood. 1998;91:3562-3565.

Duca F, Sacchi E, Tagliabue L, Tajoli E. C677T methylenetetrahydrofolate reductase (MTHFR) mutation in stroke [abstract]. Thromb Haemost. 1997;78(suppl):102.

Eikelboom JW, Hankey GJ, Anand SS, Lofthouse E, Staples N, Baker RI. Association between high homocysteine and ischemic stroke due to large- and small-artery disease but not other etiologic subtypes of ischemic stroke. Stroke. 2000;31:1069-1075.

Gaustadnes M, Rudiger N, Moller J, Rasmussen K, Bjerregaard Larsen T, Ingerslev J. Thrombophilic predisposition in stroke and venous thromboembolism in Danish patients. Blood Coagul Fibrinolysis. 1999;10(5):251-9

Gross B, Antebi A, Cassel A, Honigman S. Is a mutation in the enzyme MTHFR a risk factor for stroke in young adults [abstract]? Neurology. 2000;54(suppl 3):A142.

Harmon DL, Doyle RM, Meleady R, et al. Genetic analysis of the thermolabile variant of 5, 10-methylenetetrahydrofolate reductase as a risk factor for ischemic stroke. Arterioscler Thromb Vasc Biol. 1999;19:208-211.

Hassan A, Hunt BJ, O'Sullivan M, et al. Homocysteine is a risk factor for cerebral small vessel disease, acting via endothelial dysfunction. Brain. 2004;127(Pt 1):212-9

Hermans MP, Gala JL, Buysschaert M. The MTHFR CT polymorphism confers a high risk for stroke in both homozygous and heterozygous T allele carriers with Type 2 diabetes. Diabet Med. 2006;23(5):529-36.

Kostulas K, Crisby M, Huang WX, et al. A methylenetetrahydrofolate reductase gene polymorphism in ischaemic stroke and in carotid artery stenosis. Eur J Clin Invest. 1998;28:285-289.

Kristensen B, Malm J, Nilsson TK, et al. Hyperhomocysteinemia and hypofibrinolysis in young adults with ischemic stroke. Stroke. 1999;30:974-980.

Lalouschek W, Aull S, Serles W, et al. Genetic and nongenetic factors influencing plasma homocysteine levels in patients with ischemic cerebrovascular disease and in healthy control subjects. J Lab Clin Med. 1999;133:575-582.

Lopaciuk S, Bykowska K, Kwiecinski H, et al. Factor V Leiden, prothrombin gene G20210A variant, and methylenetetrahydrofolate reductase C677T genotype in young adults with ischemic stroke. Clin Appl Thromb Hemost. 2001;7:346-350.

Madonna P, de Stefano V, Coppola A, et al. Hyperhomocysteinemia and other inherited prothrombotic conditions in young adults with a history of ischemic stroke. Stroke. 2002;33:51-56.

Margaglione M, D'Andrea G, Giuliani N, et al. Inherited prothrombotic conditions and premature ischemic stroke: sex difference in the association with factor V Leiden. Arterioscler Thromb Vasc Biol. 1999;19:1751-1756.

Markus HS, Zhang Y, Jeffery S. Screening for the factor-V Arg 506 Gln mutation in patients with TIA and stroke. Cerebrovasc Dis. 1996;6:360-362.

McIlroy SP, Dynan KB, Lawson JT, Patterson CC, Passmore AP. Moderately elevated plasma homocysteine, methylenetetrahydrofolate reductase genotype, and risk for stroke, vascular dementia, and Alzheimer disease in Northern Ireland. Stroke. 2002;33:2351-2356.

Pezzini A, Del Zotto E, Archetti S, et al. Plasma homocysteine concentration, C677T MTHFR genotype, and 844ins68bp CBS genotype in young adults with spontaneous cervical artery dissection and atherothrombotic stroke. Stroke. 2002;33:664-669.

Pezzini A, Grassi M, Del Zotto E, et al. Interaction of homocysteine and conventional predisposing factors on risk of ischaemic stroke in young people: consistency in phenotype-disease analysis and genotype-disease analysis. J Neurol Neurosurg Psychiatry. 2006;77(10):1150-6.

Press RD, Beamer N, Evans A, DeLoughery TG, Coull BM. Role of a common mutation in the homocysteine regulatory enzyme methylenetetrahydrofolate reductase in ischemic stroke. Diagn Mol Pathol. 1999;8:54-58.

Reuner KH, Ruf A, Kaps M, Druschky KF, Patscheke H. The mutation C677→T in the methylene tetrahydrofolate reductase gene and stroke. Thromb Haemost. 1998;79:450-451.

Salooja N, Catto A, Carter A, Tudenham EG, Grant PJ. Methylene tetrahydrofolate reductase C677T genotype and stroke. Clin Lab Haematol. 1998;20:357-361.

Sanchez-Marin B, Grasa JM, Torres M, et al. Prevalence of methylenetetrahydrofolate reductase C677T mutation among patients with acute ischemic cerebrovascular disease in Aragon. An Med Interna. 2006;23(4):153-5

Sazci A, Ergul E, Tuncer N, Akpinar G, Kara I. Methylenetetrahydrofolate reductase gene polymorphisms are associated with ischemic and hemorrhagic stroke: Dual effect of MTHFR polymorphisms C677T and A1298C. Brain Res Bull. 2006;71(1-3):45-50.

Slooter AJ, Rosendaal FR, Tanis BC, Kemmeren JM, van der Graaf Y, Algra A. Prothrombotic conditions, oral contraceptives, and the risk of ischemic stroke. J Thromb Haemost. 2005;3(6):1213-7.

Soriente L, Coppola A, Madonna P, et al. Homozygous C677T mutation of the 5,10 methylenetetrahydrofolate reductase gene and hyperhomocysteinemia in Italian patients with a history of early-onset ischemic stroke. Stroke. 1998;29:869-871.

Szolnoki Z, Somogyvari F, Kondacs A, Szabo M, Fodor L, Bene J, Melegh B. Evaluation of the modifying effects of unfavourable genotypes on classical clinical risk factors for ischaemic stroke. J Neurol Neurosurg Psychiatry. 2003;74(12):1615-20.

Topic E, Timundic AM, Ttefanovic M, et al. Polymorphism of apoprotein E (APOE), methylenetetrahydrofolate reductase (MTHFR) and paraoxonase (PON1) genes in patients with cerebrovascular disease. Clin Chem Lab Med. 2001;39:346-350.

Ucar F, Sonmez M, Ovali E, et al. MTHFR C677T polymorphism and its relation to ischemic stroke in the Black Sea Turkish population. Am J Hematol. 2004;76(1):40-3.

Voetsch B, Damasceno BP, Camargo EC, et al. Inherited thrombophilia as a risk factor for the development of ischemic stroke in young adults. Thromb Haemost. 2000;83:229-233.

MTHFR Gene - Homocysteine Levels

Almawi WY, Ameen G, Tamim H, Finan RR, Irani-Hakime N. Factor V G1691A, prothrombin G20210A, and methylenetetrahydrofolate reductase [MTHFR] C677T gene polymorphism in angiographically documented coronary artery disease. J Thromb Thrombolysis. 2004;17(3):199-205.

Bailey LB, Duhaney RL, Maneval DR, et al. Vitamin B-12 status is inversely associated with plasma homocysteine in young women with C677T and/or A1298C methylenetetrahydrofolate reductase polymorphisms. J Nutr 2002; 132: 1872–78.

Baines M, Kredan MB, Usher J, Davison A, Higgins G, Taylor W, West C, Fraser WD, Ranganath LR. The association of homocysteine and its determinants MTHFR genotype, folate, vitamin B12 and vitamin B6 with bone mineral density in postmenopausal British women. Bone. 2007;40(3):730-6.

Bathum L, Petersen I, Christiansen L, Konieczna A, Sørensen TI, Kyvik KO. Genetic and environmental influences on plasma homocysteine: results from a Danish twin study. Clin Chem. 2007;53(5):971-9.

Battistelli S, Vittoria A, Stefanoni M, Bing C, Roviello F. Total plasma homocysteine and methylenetetrahydrofolate reductase C677T polymorphism in patients with colorectal carcinoma. World J Gastroenterol. 2006;12(38):6128-32.

Beagle B, Yang TL, Hung J, Cogger EA, Moriarty DJ, Caudill MA. The glycine N-methyltransferase (GNMT) 1289 C->T variant influences plasma total homocysteine concentrations in young women after restricting folate intake. J Nutr. 2005;135(12):2780-5.

Bennouar N, Allami A, Azeddoug H, Bendris A, Laraqui A, El Jaffali A, El Kadiri N, Benzidia R, Benomar A, Fellat S, Benomar M. Thermolabile Methylenetetrahydrofolate Reductase C677T Polymorphism and Homocysteine Are Risk Factors for Coronary Artery Disease in Moroccan Population. J Biomed Biotechnol. 2007;2007(1):80687.

Castro R, Rivera I, Ravasco P, et al. 5,10- Methylenetetrahydrofolate reductase 677C→T and 1298A→C mutations are genetic determinants of elevated homocysteine. QJM 2003; 96: 297–303.

Caudill MA, Le T, Moonie SA, Esfahani ST, Cogger EA. Folate status in women of childbearing age residing in Southern California after folic acid fortification. J Am Coll Nutr 2001; 20:129–34.

Chambers JC, Ireland H, Thompson E, Reilly P, Obeid OA, Refsum H, Ueland P, Lane DA, Kooner JS. Methylenetetrahydrofolate reductase 677 C-->T mutation and coronary heart disease risk in UK Indian Asians. Arterioscler Thromb Vasc Biol. 2000;20(11):2448-52.

Chango A, Boisson F, Barbe F, et al. The effect of 677C→T and 1298A→C mutations on plasma homocysteine and 5,10- methylenetetrahydrofolate reductase activity in healthy subjects. Br J Nutr 2000; 83: 593–96.

Chango A, Potier De Courcy G, et al. 5,10-methylenetetrahydrofolate reductase common mutations, folate status and plasma homocysteine in healthy French adults of the Supplementation en Vitamines et Mineraux Antioxydants (SU.VI.MAX) cohort. Br J Nutr 2000; 84: 891–96.

Christensen B, Frosst P, Lussier-Cacan S, et al. Correlation of a common mutation in the methylenetetrahydrofolate reductase gene with plasma homocysteine in patients with premature coronary artery disease. Arterioscler Thromb Vasc Biol 1997;17:569–73.

D'Angelo A, Coppola A, Madonna P, et al. The role of vitamin B12 in fasting hyperhomocysteinemia and its interaction with the homozygous C677T mutation of the methylenetetrahydrofolate reductase (MTHFR) gene: a case-control study of patients with early-onset thrombotic events. Thromb Haemost 2000; 83: 563–70.

De Bree A, Verschuren WM, Bjorke-Monsen AL, et al. Effect of the methylenetetrahydrofolate reductase 677C→T mutation on the relations among folate intake and plasma folate and homocysteine concentrations in a general population sample. Am J Clin Nutr 2003; 77: 687–93.

Dedoussis GV, Panagiotakos DB, Chrysohoou C, Pitsavos C, Zampelas A, Choumerianou D, Stefanadis C. Effect of interaction between adherence to a

Mediterranean diet and the methylenetetrahydrofolate reductase 677C-->T mutation on homocysteine concentrations in healthy adults: the ATTICA Study. Am J Clin Nutr. 2004;80(4):849-54.

Collings A, Raitakari OT, Juonala M, Rontu R, Kähönen M, Hutri-Kähönen N, Rönnemaa T, Marniemi J, Viikari JS, Lehtimäki T. Associations of methylenetetrahydrofolate reductase C677T polymorphism with markers of subclinical atherosclerosis: the Cardiovascular Risk in Young Finns Study. Scand J Clin Lab Invest. 2008;68(1):22-30.

Den Heijer M, Graafsma S, Lee SY, van Landeghem B, Kluijtmans L, Verhoef P, Beaty TH, Blom H. Homocysteine levels--before and after methionine loading-in 51 Dutch families. Eur J Hum Genet. 2005;13(6):753-62.

Dekou V, Whincup P, Papacosta O, et al. The effect of the C677T and A1298C polymorphisms in the methylenetetrahydrofolate reductase gene on homocysteine levels in elderly men and women from the British regional heart study. Atherosclerosis 2001; 154: 659–66.

Devlin AM, Clarke R, Birks J, Evans JG, Halsted CH. Interactions among polymorphisms in folate-metabolizing genes and serum total homocysteine concentrations in a healthy elderly population. Am J Clin Nutr. 2006;83(3):708-13.

DeVos L, Chanson A, Liu Z, Ciappio ED, Parnell LD, Mason JB, Tucker KL, Crott JW. Associations between single nucleotide polymorphisms in folate uptake and metabolizing genes with blood folate, homocysteine, and DNA uracil concentrations. Am J Clin Nutr. 2008;88(4):1149-58.

Dikmen M, Ozbabalik D, Gunes HV, Degirmenci I, Bal C, Ozdemir G, Basaran A. Acute stroke in relation to homocysteine and methylenetetrahydrofolate reductase gene polymorphisms. Acta Neurol Scand. 2006;113(5):307-14.

Domagala TB, Adamek L, Nizankowska E, Sanak M, Szczeklik A. Mutations C677T and A1298C of the 5,10-methylenetetrahydrofolate reductase gene and fasting plasma homocysteine levels are not associated with the increased risk of venous thromboembolic disease. Blood Coagul Fibrinol 2002; 13: 423–31.

Fohr IP, Prinz-Langenohl R, Bronstrup A, et al. 5,10-Methylenetetrahydrofolate reductase genotype determines the plasma homocysteine-lowering effect of supplementation with 5-methyltetrahydrofolate or folic acid in healthy young women. Am J Clin Nutr 2002; 75: 275–82.

Frederiksen J, Juul K, Grande P, Jensen GB, Schroeder TV, Tybjaerg-Hansen A, Nordestgaard BG. Methylenetetrahydrofolate reductase polymorphism (C677T), hyperhomocysteinemia, and risk of ischemic cardiovascular disease and venous thromboembolism: prospective and case-control studies from the Copenhagen City Heart Study. Blood. 2004;104(10):3046-51.

Freitas AI, Mendonça I, Guerra G, Brión M, Reis RP, Carracedo A, Brehm A. Methylenetetrahydrofolate reductase gene, homocysteine and coronary artery disease: the A1298C polymorphism does matter. Inferences from a case study (Madeira, Portugal). Thromb Res. 2008;122(5):648-56.

Friedman G, Goldschmidt N, Friedlander Y, et al. A common mutation A1298C in human methylenetetrahydrofolate reductase gene: association with plasma total homocysteine and folate concentrations. J Nutr 1999; 129: 1656–61.

Gasparovic J, Raslová K, Basistová Z, Zacharová M, Wsólová L, Avdicová M, Blazícek P, Lietava J, Siváková D. Effect of C677T methylenetetrahydrofolate reductase gene polymorphism on plasma homocysteine levels in ethnic groups. Physiol Res. 2004;53(2):215-8.

Gemmati D, Previati M, Serino ML, et al. Low folate levels and thermolabile methylenetetrahydrofolate reductase as primary determinant of mild hyperhomocystinemia in normal and thromboembolic subjects. Arterioscler Thromb Vasc Biol 1999; 19:1761–67.

Giusti B, Gori AM, Marcucci R, Sestini I, Saracini C, Sticchi E, Gensini F, Fatini C, Abbate R, Gensini GF. Role of C677T and A1298C MTHFR, A2756G MTR and -786 C/T eNOS gene polymorphisms in atrial fibrillation susceptibility. PLoS One. 2007;2(6):e495.

Gjesdal CG, Vollset SE, Ueland PM, Refsum H, Meyer HE, Tell GS. Plasma homocysteine, folate, and vitamin B 12 and the risk of hip fracture: the hordaland homocysteine study. J Bone Miner Res. 2007;22(5):747-56.

Golbahar J, Hamidi A, Aminzadeh MA, Omrani GR. Association of plasma folate, plasma total homocysteine, but not methylenetetrahydrofolate reductase C667T polymorphism, with bone mineral density in postmenopausal Iranian women: a cross sectional study. Bone. 2004;35(3):760-5.

Guinotte CL, Burns MG, Axume JA, et al. Methylenetetrahydrofolate reductase 677C→T variant modulates folate status response to controlled folate intakes in young women. J Nutr 2003; 133: 1272–80.

Husemoen LL, Thomsen TF, Fenger M, Jorgensen HL, Jorgensen T. Contribution of thermolabile methylenetetrahydrofolate reductase variant to total plasma homocysteine levels in healthy men and women. Genet Epidemiol 2003; 24: 322–30.

Hustad S, Ueland PM, Vollset SE, Zhang Y, Bjorke-Monsen AL, Schneede J. Riboflavin as a determinant of plasma total homocysteine: effect modification by the methylenetetrahydrofolate reductase C677T polymorphism. Clin Chem 2000; 46: 1065–71.

Ilhan N, Kucuksu M, Kaman D, Ilhan N, Ozbay Y. The 677 C/T MTHFR polymorphism is associated with essential hypertension, coronary artery disease, and higher homocysteine levels. Arch Med Res. 2008;39(1):125-30.

Jacques PF, Kalmbach R, Bagley PJ, et al. The relationship between riboflavin and plasma total homocysteine in the Framingham Offspring cohort is influenced by folate status and the C677T transition in the methylenetetrahydrofolate reductase gene. J Nutr 2002; 132: 283–88.

Kölling K, Ndrepepa G, Koch W, Braun S, Mehilli J, Schömig A, Kastrati A. Methylenetetrahydrofolate reductase gene C677T and A1298C polymorphisms, plasma homocysteine, folate, and vitamin B12 levels and the extent of coronary artery disease. Am J Cardiol. 2004;93(10):1201-6.

Litynski P, Loehrer F, Linder L, Todesco L, Fowler B. Effect of low doses of 5-methyltetrahydrofolate and folic acid on plasma homocysteine in healthy subjects with or without the 677C→T polymorphism of methylenetetrahydrofolate reductase. Eur J Clin Invest 2002; 32: 662–68.

Lopreato FR, Stabler SP, Carvalho FR, Hirata RD, Hirata MH, Robi DL, Sampaio-Neto LF, Allen RH, Guerra-Shinohara EM. Relationships between gene polymorphisms of folate-related proteins and vitamins and metabolites in pregnant women and neonates. Clin Chim Acta. 2008;398(1-2):134-9.

Ma J, Stampfer MJ, Hennekens CH, et al. Methylenetetrahydrofolate reductase polymorphism, plasma folate, homocysteine, and risk of myocardial infarction in US physicians. Circulation 1996; 94: 2410–16.

McLean RR, Karasik D, Selhub J, Tucker KL, Ordovas JM, Russo GT, Cupples LA, Jacques PF, Kiel DP. Association of a common polymorphism in the

methylenetetrahydrofolate reductase (MTHFR) gene with bone phenotypes depends on plasma folate status. J Bone Miner Res. 2004;19(3):410-8.

McNulty H, Dowey le RC, Strain JJ, Dunne A, Ward M, Molloy AM, McAnena LB, Hughes JP, Hannon-Fletcher M, Scott JM. Riboflavin lowers homocysteine in individuals homozygous for the MTHFR 677C->T polymorphism. Circulation. 2006 3;113(1):74-80.

Meisel C, Cascorbi I, Gerloff T, et al. Identification of six methylenetetrahydrofolate reductase (MTHFR) genotypes resulting from common polymorphisms: impact on plasma homocysteine levels and development of coronary artery disease. Atherosclerosis 2001; 154: 651–58.

Meleady R, Ueland PM, Blom H, et al. Thermolabile methylenetetrahydrofolate reductase, homocysteine, and cardiovascular disease risk: the European Concerted Action Project. Am J Clin Nutr 2003; 77: 63–70.

Mtiraoui N, Ezzidi I, Chaieb M, Marmouche H, Aouni Z, Chaieb A, Mahjoub T, Vaxillaire M, Almawi WY. MTHFR C677T and A1298C gene polymorphisms and hyperhomocysteinemia as risk factors of diabetic nephropathy in type 2 diabetes patients. Diabetes Res Clin Pract. 2007;75(1):99-106.

Naess IA, Christiansen SC, Romundstad PR, Cannegieter SC, Blom HJ, Rosendaal FR, Hammerstrøm J. Prospective study of homocysteine and MTHFR 677TT genotype and risk for venous thrombosis in a general population--results from the HUNT 2 study. Br J Haematol. 2008;141(4):529-35.

Narayanan S, McConnell J, Little J, Sharp L, Piyathilake CJ, Powers H, Basten G, Duthie SJ. Associations between two common variants C677T and A1298C in the methylenetetrahydrofolate reductase gene and measures of folate metabolism and DNA stability (strand breaks, misincorporated uracil, and DNA methylation status)

in human lymphocytes in vivo. Cancer Epidemiol Biomarkers Prev. 2004;13(9):1436-43.

Ozbek Z, Kucukali CI, Ozkok E, Orhan N, Aydin M, Kilic G, Sazci A, Kara I. Effect of the methylenetetrahydrofolate reductase gene polymorphisms on homocysteine, folate and vitamin B12 in patients with bipolar disorder and relatives. Prog Neuropsychopharmacol Biol Psychiatry. 2008;32(5):1331-7.

Passaro A, Vanini A, Calzoni F, et al. Plasma homocysteine, methylenetetrahydrofolate reductase mutation and carotid damage in elderly healthy women. Atherosclerosis 2001; 157: 175–80.

Pullin CH, Ashfield-Watt PA, Burr ML, et al. Optimization of dietary folate or low-dose folic acid supplements lower homocysteine but do not enhance endothelial function in healthy adults, irrespective of the methylenetetrahydrofolate reductase (C677T) genotype. J Am Coll Cardiol 2001; 38:1799–805.

Reyes-Engel A, Munoz E, Gaitan MJ, et al. Implications on human fertility of the 677C→T and 1298A→C polymorphisms of the MTHFR gene: consequences of a possible genetic selection. Mol Hum Reprod 2002; 8: 952–57.

Schmitz C, Lindpaintner K, Verhoef P, Gaziano JM, Buring J. Genetic polymorphism of methylenetetrahydrofolate reductase and myocardial infarction: a case-control study. Circulation 1996; 94: 1812–14.

Schwartz SM, Siscovick DS, Malinow MR, et al. Myocardial infarction in young women in relation to plasma total homocysteine, folate, and a common variant in the methylenetetrahydrofolate reductase gene. Circulation 1997; 96:412–17.

Shelnutt KP, Kauwell GP, Chapman CM, Gregory JF 3rd, Maneval DR, Browdy AA,

Theriaque DW, Bailey LB. Folate status response to controlled folate intake is affected by the methylenetetrahydrofolate reductase 677C-->T polymorphism in young women. J Nutr. 2003;133(12):4107-11.

Silaste ML, Rantala M, Sampi M, Alfthan G, Aro A, Kesaniemi YA. Polymorphisms of key enzymes in homocysteine metabolism affect diet responsiveness of plasma homocysteine in healthy women. J Nutr 2001; 131: 2643–47.

Thogersen AM, Nilsson TK, Dahlen G, et al. Homozygosity for the C677→T mutation of 5,10-methylenetetrahydrofolate reductase and total plasma homocysteine are not associated with greater than normal risk of a first myocardial infarction in northern Sweden. Coron Artery Dis 2001; 12: 85–90.

Thuillier L, Chadefaux-Vekemans B, Bonnefont JP, et al. Does the polymorphism 677C T of the 5,10-methylenetetrahydrofolate reductase gene contribute to homocysteine related vascular disease? J Inherit Metab Dis 1998; 21: 812–22.

Ulvik A, Ueland PM, Fredriksen A, Meyer K, Vollset SE, Hoff G, Schneede J. Functional inference of the methylenetetrahydrofolate reductase 677C > T and 1298A > C polymorphisms from a large-scale epidemiological study. Hum Genet. 2007;121(1):57-64.

Varela ML, Adamczuk YP, Forastiero RR, et al. Major and potential prothrombotic genotypes in a cohort of patients with venous thromboembolism. Thromb Res 2001; 104: 317–24.

Vaughn JD, Bailey LB, Shelnutt KP, Dunwoody KM, Maneval DR, Davis SR, Quinlivan EP, Gregory JF 3rd, Theriaque DW, Kauwell GP. Methionine synthase reductase 66A->G polymorphism is associated with increased plasma homocysteine concentration when combined with the homozygous methylenetetrahydrofolate reductase 677C->T variant. J Nutr. 2004;134(11):2985-90.

Vollset SE, Igland J, Jenab M, Fredriksen A, Meyer K, et al. The association of gastric cancer risk with plasma folate, cobalamin, and methylenetetrahydrofolate reductase polymorphisms in the European Prospective Investigation into Cancer and Nutrition. Cancer Epidemiol Biomarkers Prev. 2007;16(11):2416-24.

Voutilainen S, Lakka TA, Hamelahti P, Lehtimaki T, Poulsen HE, Salonen JT. Plasma total homocysteine concentration and the risk of acute coronary events: the Kuopio Ischaemic Heart Disease Risk Factor Study. J Intern Med 2000; 248: 217–22.

Yang QH, Botto LD, Gallagher M, Friedman JM, Sanders CL, Koontz D, Nikolova S, Erickson JD, Steinberg K. Prevalence and effects of gene-gene and gene-nutrient interactions on serum folate and serum total homocysteine concentrations in the United States: findings from the third National Health and Nutrition Examination Survey DNA Bank. Am J Clin Nutr. 2008;88(1):232-46.

Zee RY, Mora S, Cheng S, Erlich HA, Lindpaintner K, Rifai N, Buring JE, Ridker PM. Homocysteine, 5,10-methylenetetrahydrofolate reductase 677C>T polymorphism, nutrient intake, and incident cardiovascular disease in 24,968 initially healthy women. Clin Chem. 2007;53(5):845-51.

Zittoun J, Tonetti C, Bories D, Pignon JM, Tulliez M. Plasma homocysteine levels related to interactions between folate status and methylenetetrahydrofolate reductase: a study in 52 healthy subjects. Metabolism 1998; 47: 1413–18.

<u>Homocysteine Levels - Stroke</u>

Wald DS, Law M, Morris JK. Homocysteine and cardiovascular disease: evidence on causality from a meta-analysis. BMJ 2002; 325: 1202.

<u>PROTHROMBIN</u>

Prothrombin Gene – Stroke

Aznar J, Mira Y, Vaya A, et al. Factor V Leiden and prothrombin G20210A mutations in young adults with cryptogenic ischemic stroke. Thromb Haemost. 2004;91(5):1031-4

Bentolila S, Ripoll L, Drouet L, Mazoyer E, Woimant F. Thrombophilia due to 20210 G→A prothrombin polymorphism and cerebral ischemia in the young. Stroke. 1997;28:1846-1847.

Botto N, Spadoni I, Giusti S, Ait-Ali L, Sicari R, Andreassi MG. Prothrombotic mutations as risk factors for cryptogenic ischemic cerebrovascular events in young subjects with patent foramen ovale. Stroke. 2007;38(7):2070-3.

De Stefano V, Chiusolo P, Paciaroni K, et al. Prothrombin G20210A mutant genotype is a risk factor for cerebrovascular ischemic disease in young patients. Blood. 1998;91:3562-3565.

Egan RA, Kuyl JM, Press R, Lutsep HL. Lack of prothrombin gene mutation in young stroke patients. J Stroke Cerebrovasc Dis. 2000;9:229-231.

Eterović D, Titlić M, Culić V, Zadro R, Primorac D. Lower contribution of factor V Leiden or G202104 mutations to ischemic stroke in patients with clinical risk factors: pair-matched case-control study. Clin Appl Thromb Hemost. 2007;13(2):188-93.

Gomez Garcia EB, van Goor MP, Leebeek FW, Brouwers GJ, Koudstaal PJ, Dippel DW. Elevated prothrombin is a risk factor for cerebral arterial ischemia in young adults. Clin Neurol Neurosurg. 2002;104:285-288.

Halbmayer WM, Haushofer A, Hermann KM, Fischer M. The 20210A allele of the prothrombin gene: a risk factor for juvenile stroke? result of a pilot study [letter]. Blood Coagul Fibrinolysis. 1998;9:209-210.

Hankey GJ, Eikelboom JW, van Bockxmeer FM, Lofthouse E, Staples N, Baker RI. Inherited thrombophilia in ischemic stroke and its pathogenic subtypes. Stroke. 2001;32:1793-1799.

Iniesta JA, Corral J, Gonzalez-Conejero R, Rivera J, Vicente V. Prothrombotic genetic risk factors in patients with coexisting migraine and ischemic cerebrovascular disease. Headache. 1999;39:486-489.

Lalouschek W, Aull S, Series W, Zeiler K, Mannhalter C. The prothrombin G20210A mutation and factor V Leiden mutation in patients with cerebrovascular disease [letter]. Blood. 1998;92:704-705.

Lalouschek W, Schillinger M, Hsieh K, et al. Matched case-control study on factor V Leiden and the prothrombin G20210A mutation in patients with ischemic stroke/transient ischemic attack up to the age of 60 years. Stroke. 2005;36(7):1405-9

Lichy C, Reuner KH, Buggle F, et al. Prothrombin g20210a mutation, but not factor V Leiden, is a risk factor in patients with cerebral ischemia associated with persistent foramen ovale. Paper presented at: 16th International Congress on Fibrinolysis and Proteolysis in conjunction with the 17th International Fibrinogen Workshop; September 8-13, 2002; Munich, Germany.

Lopaciuk S, Bykowska K, Kwiecinski H, et al. Factor V Leiden, prothrombin gene G20210A variant, and methylenetetrahydrofolate reductase C677T genotype in young adults with ischemic stroke. Clin Appl Thromb Hemost. 2001;7:346-350.

Madonna P, de Stefano V, Coppola A, et al. Hyperhomocysteinemia and other inherited prothrombotic conditions in young adults with a history of ischemic stroke. Stroke. 2002;33:51-56.

Margaglione M, D'Andrea G, Giuliani N, et al. Inherited prothrombotic conditions and premature ischemic stroke: sex difference in the association with factor V Leiden. Arterioscler Thromb Vasc Biol. 1999;19:1751-1756.

Martinelli I, Franchi F, Akwan S, Bettini P, Merati G, Mannucci PM. The transition G to A at position 20210 in the 3'-untranslated region of the prothrombin gene is not associated with cerebral ischemia [letter]. Blood. 1997;90:3806.

Meseguer E, Llamas P, Fernandez de Velasco J, et al. Prothrombotic factors in stroke (Spanish). Neurologia. 2004;19(3):99-105

Pezzini A, Grassi M, Del Zotto E, et al. Cumulative effect of predisposing genotypes and their interaction with modifiable factors on the risk of ischemic stroke in young adults. Stroke. 2005;36(3):533-9

Reuner KH, Ruf A, Grau A, et al. Prothrombin gene G20210→A transition is a risk factor for cerebral venous thrombosis. Stroke. 1998;29:1765-1769.

Ridker PM, Hennekens CH, Miletich JP. G20210A mutation in prothrombin gene and risk of myocardial infarction, stroke, and venous thrombosis in a large cohort of US men. Circulation. 1999;99:999-1004.

Smiles AM, Jenny NS, Tang Z, Arnold A, Cushman M, Tracy RP. No association of plasma prothrombin concentration or the G20210A mutation with incident cardiovascular disease: results from the Cardiovascular Health Study. Thromb Haemost. 2002;87:614-621.

Szolnoki Z, Somogyvari F, Kondacs A, Szabo M, Fodor L, Bene J, Melegh B. Evaluation of the modifying effects of unfavourable genotypes on classical clinical risk factors for ischaemic stroke. J Neurol Neurosurg Psychiatry. 2003;74(12):1615-20.

Voetsch B, Damasceno BP, Camargo EC, et al. Inherited thrombophilia as a risk factor for the development of ischemic stroke in young adults. Thromb Haemost. 2000;83:229-233.

<u>Prothrombin Gene – Prothrombin Levels</u>

Balasa VV, Gruppo RA, Glueck CJ, Stroop D, Becker A, Pillow A, Wang P. The relationship of mutations in the MTHFR, prothrombin, and PAI-1 genes to plasma levels of homocysteine, prothrombin, and PAI-1 in children and adults. Thromb Haemost. 1999;81(5):739-44.

Castoldi E, Simioni P, Tormene D, Thomassen MC, Spiezia L, Gavasso S, Rosing J. Differential effects of high prothrombin levels on thrombin generation depending on the cause of the hyperprothrombinemia. J Thromb Haemost. 2007;5(5):971-9.

Chinthammitr Y, Vos HL, Rosendaal FR, Doggen CJ. The association of prothrombin A19911G polymorphism with plasma prothrombin activity and venous thrombosis: results of the MEGA study, a large population-based case-control study. J Thromb Haemost. 2006;4(12):2587-92.

Colucci M, Binetti BM, Tripodi A, Chantarangkul V, Semeraro N. Hyperprothrombinemia associated with prothrombin G20210A mutation inhibits plasma fibrinolysis through a TAFI-mediated mechanism. Blood. 2004;103(6):2157-61.

Ferraresi P, Marchetti G, Legnani C, Cavallari E, Castoldi E, Mascoli F, Ardissino D, Palareti G, Bernardi F. The heterozygous 20210 G/A prothrombin genotype is associated

with early venous thrombosis in inherited thrombophilias and is not increased in frequency in artery disease. Arterioscler Thromb Vasc Biol. 1997;17(11):2418-22.

Folsom AR, Cushman M, Tsai MY, Heckbert SR, Aleksic N. Prospective study of the G20210A polymorphism in the prothrombin gene, plasma prothrombin concentration, and incidence of venous thromboembolism. Am J Hematol. 2002;71(4):285-90.

Gruenewald M, Germowitz A, Beneke H, Guethner C, Griesshammer M. Coagulation factor II activity determination is not useful as a screening tool for the G20210A prothrombin gene allele. Thromb Haemost. 2000;84(1):141-2.

Koenen RR, Tans G, van Oerle R, Hamulyák K, Rosing J, Hackeng TM. The APC-independent anticoagulant activity of protein S in plasma is decreased by elevated prothrombin levels due to the prothrombin G20210A mutation. Blood. 2003;102(5):1686-92.

Kyrle PA, Mannhalter C, Béguin S, Stümpflen A, Hirschl M, Weltermann A, Stain M, Brenner B, Speiser W, Pabinger I, Lechner K, Eichinger S. Clinical studies and thrombin generation in patients homozygous or heterozygous for the G20210A mutation in the prothrombin gene. Arterioscler Thromb Vasc Biol. 1998;18(8):1287-91.

Poort SR, Rosendaal FR, Reitsma PH, Bertina RM. A common genetic variation in the 3'-untranslated region of the prothrombin gene is associated with elevated plasma prothrombin levels and an increase in venous thrombosis. Blood. 1996;88(10):3698-703.

Reiner AP, Carlson CS, Rieder MJ, Schwartz SM, Siscovick DS. Common genomic sequence variation of the prothrombin gene and risk of non-fatal myocardial infarction in white women. J Thromb Haemost. 2005;3(12):2809-11.

Simioni P, Tormene D, Manfrin D, Gavasso S, Luni S, Stocco D, Girolami A. Prothrombin antigen levels in symptomatic and asymptomatic carriers of the 20210A prothrombin variant. Br J Haematol. 1998;103(4):1045-50.

Soria JM, Almasy L, Souto JC, Tirado I, Borell M, Mateo J, Slifer S, Stone W, Blangero J, Fontcuberta J. Linkage analysis demonstrates that the prothrombin G20210A mutation jointly influences plasma prothrombin levels and risk of thrombosis. Blood. 2000;95(9):2780-5.

Souto JC, Mateo J, Soria JM, Llobet D, Coll I, Borrell M, Fontcuberta J. Homozygotes for prothrombin gene 20210 A allele in a thrombophilic family without clinical manifestations of venous thromboembolism. Haematologica. 1999;84(7):627-32.

von Ahsen N, Lewczuk P, Schütz E, Oellerich M, Ehrenreich H. Prothrombin activity and concentration in healthy subjects with and without the prothrombin G20210A mutation. Thromb Res. 2000;99(6):549-56.

Prothrombin Levels – Stroke

Smiles AM, Jenny NS, Tang Z, Arnold A, Cushman M, Tracy RP. No association of plasma prothrombin concentration or the G20210A mutation with incident cardiovascular disease: results from the Cardiovascular Health Study. Thromb Haemost. 2002;87(4):614-21.

Gómez Garcia EB, van Goor MP, Leebeek FW, Brouwers GJ, Koudstaal PJ, Dippel DW. Elevated prothrombin is a risk factor for cerebral arterial ischemia in young adults. Clin Neurol Neurosurg. 2002;104(4):285-8.

PAI-1

PAI-1 Gene – Stroke

Adamski MG, Turaj W, Slowik A, Wloch-Kopec D, Wolkow P, Szczudlik A. A-G-4G haplotype of PAI-1 gene polymorphisms -844 G/A, HindIII G/C, and -675 4G/5G is associated with increased risk of ischemic stroke caused by small vessel disease. Acta Neurol Scand. 2009;120(2):94-100.

Attia J, Thakkinstian A, Wang Y, et al. The PAI-1 4G/5G Gene Polymorphism and Ischemic Stroke: An Association Study and Meta-Analysis. J Stroke Cerebrovas Dis. 2007;16(4)173-179

Balta G, Altay C, Gurgey A. PAI-1 gene 4G/5G genotype: A risk factor for thrombosis in vessels of internal organs. Am J Hematol. 2002;71(2):89-93.

Catto AJ, Kohler HP, Bannan S, Stickland M, Carter A, Grant PJ. Factor XIII Val 34 Leu: a novel association with primary intracerebral hemorrhage. Stroke. 1998;29:813-816.

Elbaz A, Cambien F, Amarenco P; GENIC investigators. Plasminogen activator inhibitor genotype and brain infarction. Circulation. 2001;103(2):e13-4

Endler G, Lalouschek W, Exner M, Mitterbauer G, Haring D, Mannhalter C. The 4G/4G genotype at nucleotide position –675 in the promotor region of the plasminogen activator inhibitor 1 (PAI–1) gene is less frequent in young patients with minor stroke than in controls. Br J Haematol. 2000;110:469-471.

Hindorff LA, Schwartz SM, Siscovick DS, Psaty BM, Longstreth WT Jr, Reiner AP. The association of PAI-1 promoter 4G/5G insertion/deletion polymorphism with myocardial infarction and stroke in young women. J Cardiovasc Risk. 2002;9:131-137.

Hoekstra T, Geleijnse JM, de Waart F, Nederhand R, Kluft C, Kok FJ, Schouten EG. The 4G/5G-polymorphism in the PAI-1 gene is not associated with markers of atherosclerosis in male smokers. Thromb Res. 2002;107(3-4):115-9.

Jood K, Ladenvall P, Tjarnlund-Wolf A, et al. Fibrinolytic gene polymorphism and ischemic stroke. Stroke. 2005;36(10):2077-81.

Kucukarabaci B, Gunes HV, Ozdemir G, et al. Investigation of association between plasminogen activator inhibitor type-1 (PAI-1) gene 4G/5G polymorphism frequency and plasma PAI-1 enzyme activity in patients with acute stroke. Genet Test. 2008;12(3):443-51.

Saidi S, Slamia LB, Mahjoub T, Ammou SB, Almawi WY. Association of PAI-1 4G/5G and -844G/A gene polymorphism and changes in PAI-1/tPA levels in stroke: a case-control study. J Stroke Cerebrovasc Dis. 2007;16(4):153-9.

van Goor ML, Gómez García E, Brouwers GJ, Leebeek FW, Koudstaal PJ, Dippel DW. PLA1/A2 polymorphism of the platelet glycoprotein receptor IIb/IIIa in young patients with cryptogenic TIA or ischemic stroke. Thrombosis Research. 2002;108:63-65

van Goor ML, Garcia EG, Leebeek F, Brouwers GJ, Koudstaal P, Dippel D. The plasminogen activator inhibitor (PAI-1) 4G/5G promoter polymorphism and PAI-1 levels in ischemic stroke. A case-control study. Thromb Haemost. 2005;93(1):92-6.

Wiklund PG, Nilsson L, Ardnor SN, et al. Plasminogen activator inhibitor-1 4G/5G polymorphism and risk of stroke: replicated findings in two nested case-control studies based on independent cohorts. Stroke. 2005;36(8):1661-5

<u>PAI-1 Gene – PAI-1 Levels</u>

Balasa VV, Gruppo RA, Glueck CJ, Stroop D, Becker A, Pillow A, Wang P. The relationship of mutations in the MTHFR, prothrombin, and PAI-1 genes to plasma levels of homocysteine, prothrombin, and PAI-1 in children and adults. Thromb Haemost. 1999;81(5):739-44.

Bonfigli AR, Sirolla C, Cenerelli S, Marra M, Boemi M, Franceschi C, Testa I, Mari D, Sacchi E, Testa R. Plasminogen activator inhibitor-1 plasma level increases with age in subjects with the 4G allele at position -675 in the promoter region. Thromb Haemost. 2004;92(5):1164-5.

Burzotta F, Di Castelnuovo A, Amore C, D'Orazio A, Di Bitondo R, Donati MB, Iacoviello L. 4G/5G promoter PAI-1 gene polymorphism is associated with plasmatic PAI-1 activity in Italians: a model of gene-environment interaction. Thromb Haemost. 1998;79(2):354-8.

Diamanti-Kandarakis E, Palioniko G, Alexandraki K, Bergiele A, Koutsouba T, Bartzis M. The prevalence of 4G5G polymorphism of plasminogen activator inhibitor-1 (PAI-1) gene in polycystic ovarian syndrome and its association with plasma PAI-1 levels. Eur J Endocrinol. 2004;150(6):793-8.

Ding J, Nicklas BJ, Fallin MD, de Rekeneire N, Kritchevsky SB, Pahor M, Rodondi N, Li R, Zmuda JM, Harris TB. Plasminogen activator inhibitor type 1 gene polymorphisms and haplotypes are associated with plasma plasminogen activator inhibitor type 1 levels but not with myocardial infarction or stroke. Am Heart J. 2006;152(6):1109-15.

Doggen CJ, Bertina RM, Cats VM, Reitsma PH, Rosendaal FR. The 4G/5G polymorphism in the plasminogen activator inhibitor-1 gene is not associated with myocardial infarction. Thromb Haemost. 1999;82(1):115-20.

Eriksson P, Kallin B, van 't Hooft FM, Båvenholm P, Hamsten A. Allele-specific increase in basal transcription of the plasminogen-activator inhibitor 1 gene is associated with myocardial infarction. Proc Natl Acad Sci U S A. 1995;92(6):1851-5.

Festa A, D'Agostino R Jr, Rich SS, Jenny NS, Tracy RP, Haffner SM. Promoter (4G/5G) plasminogen activator inhibitor-1 genotype and plasminogen activator inhibitor-1 levels in blacks, Hispanics, and non-Hispanic whites: the Insulin Resistance Atherosclerosis Study. Circulation. 2003;107(19):2422-7.

Grancha S, Estellés A, Tormo G, Falco C, Gilabert J, España F, Cano A, Segui R, Aznar J. Plasminogen activator inhibitor-1 (PAI-1) promoter 4G/5G genotype and increased PAI-1 circulating levels in postmenopausal women with coronary artery disease. Thromb Haemost. 1999;81(4):516-21.

Henry M, Tregouët DA, Alessi MC, Aillaud MF, Visvikis S, Siest G, Tiret L, Juhan-Vague I. Metabolic determinants are much more important than genetic polymorphisms in determining the PAI-1 activity and antigen plasma concentrations: a family study with part of the Stanislas Cohort. Arterioscler Thromb Vasc Biol. 1998;18(1):84-91.

Hoekstra T, Geleijnse JM, Schouten EG, Kluft C. Diurnal variation in PAI-1 activity predominantly confined to the 4G-allele of the PAI-1 gene. Thromb Haemost. 2002;88(5):794-8.

Jastrzebska M, Goracy I, Naruszewicz M. Relationships between fibrinogen, plasminogen activator inhibitor-1, and their gene polymorphisms in current smokers with essential hypertension. Thromb Res. 2003;110(5-6):339-44.

Kathiresan S, Gabriel SB, Yang Q, Lochner AL, Larson MG, Levy D, Tofler GH, Hirschhorn JN, O'Donnell CJ. Comprehensive survey of common genetic variation at the plasminogen activator inhibitor-1 locus and relations to circulating plasminogen activator inhibitor-1 levels. Circulation. 2005;112(12):1728-35.

Kucukarabaci B, Gunes HV, Ozdemir G, et al. Investigation of association between plasminogen activator inhibitor type-1 (PAI-1) gene 4G/5G polymorphism frequency and plasma PAI-1 enzyme activity in patients with acute stroke. Genet Test. 2008;12(3):443-51.

Leander K, Wiman B, Hallqvist J, Sten-Linder M, de Faire U; Stockholm Heart Epidemiology Program. PAI-1 level and the PAI-1 4G/5G polymorphism in relation to risk of non-fatal myocardial infarction: results from the Stockholm Heart Epidemiology Program (SHEEP). Thromb Haemost. 2003;89(6):1064-71.

Margaglione M, Grandone E, Vecchione G, Cappucci G, Giuliani N, Colaizzo D, Celentano E, Panico S, Di Minno G. Plasminogen activator inhibitor-1 (PAI-1) antigen plasma levels in subjects attending a metabolic ward: relation to polymorphisms of PAI-1 and angiontensin converting enzyme (ACE) genes. Arterioscler Thromb Vasc Biol. 1997;17(10):2082-7.

Margaglione M, Cappucci G, d'Addedda M, Colaizzo D, Giuliani N, Vecchione G, Mascolo G, Grandone E, Di Minno G. PAI-1 plasma levels in a general population without clinical evidence of atherosclerosis: relation to environmental and genetic determinants. Arterioscler Thromb Vasc Biol. 1998;18(4):562-7.

Naran NH, Chetty N, Crowther NJ. The influence of metabolic syndrome components on plasma PAI-1 concentrations is modified by the PAI-1 4G/5G genotype and ethnicity. Atherosclerosis. 2008;196(1):155-63.

Pérez-Martínez P, Adarraga-Cansino MD, Fernández de la Puebla RA, Blanco-Molina A, Delgado-Lista J, Marín C, Ordovás JM, López-Miranda J, Pérez-Jiménez F. The -675 4G/5G polymorphism at the Plasminogen Activator Inhibitor 1 (PAI-1) gene modulates plasma Plasminogen Activator Inhibitor 1 concentrations in response to dietary fat consumption. Br J Nutr. 2008;99(4):699-702.

Saidi S, Slamia LB, Mahjoub T, Ammou SB, Almawi WY. Association of PAI-1 4G/5G and -844G/A gene polymorphism and changes in PAI-1/tPA levels in stroke: a case-control study. J Stroke Cerebrovasc Dis. 2007;16(4):153-9.

Stegnar M, Uhrin P, Peternel P, Mavri A, Salobir-Pajnic B, Stare J, Binder BR. The 4G/5G sequence polymorphism in the promoter of plasminogen activator inhibitor-1 (PAI-1) gene: relationship to plasma PAI-1 level in venous thromboembolism. Thromb Haemost. 1998;79(5):975-9.

Väisänen SB, Humphries SE, Luong LA, Penttilä I, Bouchard C, Rauramaa R. Regular exercise, plasminogen activator inhibitor-1 (PAI-1) activity and the 4G/5G promoter polymorphism in the PAI-1 gene. Thromb Haemost. 1999;82(3):1117-20.

Verschuur M, Jellema A, Bladbjerg EM, M Feskens EJ, Mensink RP, Møller L, Vos HL, de Maat MP. The plasminogen activator inhibitor-1 (PAI-1) promoter haplotype is related to PAI-1 plasma concentrations in lean individuals. Atherosclerosis. 2005;181(2):275-84.

PAI-1 Levels - Stroke

Saidi S, Slamia LB, Mahjoub T, Ammou SB, Almawi WY. Association of PAI-1 4G/5G and -844G/A gene polymorphism and changes in PAI-1/tPA levels in stroke: a case-control study. J Stroke Cerebrovasc Dis. 2007;16(4):153-9.

Jood K, Ladenvall P, Tjarnlund-Wolf A, et al. Fibrinolytic gene polymorphism and ischemic stroke. Stroke. 2005;36(10):2077-81.

Kristensen B, Malm J, Nilsson TK, Hultdin J, Carlberg B, Olsson T. Increased fibrinogen levels and acquired hypofibrinolysis in young adults with ischemic stroke. Stroke. 1998;29(11):2261-7.

Smith A, Patterson C, Yarnell J, Rumley A, Ben-Shlomo Y, Lowe G. Which hemostatic markers add to the predictive value of conventional risk factors for coronary heart disease and ischemic stroke? The Caerphilly Study. Circulation. 2005;112(20):3080-7.

van Goor ML, Garcia EG, Leebeek F, Brouwers GJ, Koudstaal P, Dippel D. The plasminogen activator inhibitor (PAI-1) 4G/5G promoter polymorphism and PAI-1 levels in ischemic stroke. A case-control study. Thromb Haemost. 2005;93(1):92-6.

PAI-1 Gene – tPA Levels

Brown NJ, Murphey LJ, Srikuma N, Koschachuhanan N, Williams GH, Vaughan DE. Interactive effect of PAI-1 4G/5G genotype and salt intake on PAI-1 antigen. Arterioscler Thromb Vasc Biol. 2001;21(6):1071-7.

Burzotta F, Di Castelnuovo A, Amore C, D'Orazio A, Di Bitondo R, Donati MB, Iacoviello L. 4G/5G promoter PAI-1 gene polymorphism is associated with plasmatic PAI-1 activity in Italians: a model of gene-environment interaction. Thromb Haemost. 1998;79(2):354-8.

Henry M, Tregouët DA, Alessi MC, Aillaud MF, Visvikis S, Siest G, Tiret L, Juhan-Vague I. Metabolic determinants are much more important than genetic polymorphisms in determining the PAI-1 activity and antigen plasma concentrations: a family study with part of the Stanislas Cohort. Arterioscler Thromb Vasc Biol. 1998;18(1):84-91.

Hoekstra T, Geleijnse JM, Schouten EG, Kluft C. Diurnal variation in PAI-1 activity predominantly confined to the 4G-allele of the PAI-1 gene. Thromb Haemost. 2002;88(5):794-8.

Jastrzebska M, Goracy I, Naruszewicz M. Relationships between fibrinogen, plasminogen activator inhibitor-1, and their gene polymorphisms in current smokers with essential hypertension. Thromb Res. 2003;110(5-6):339-44.

Margaglione M, Grandone E, Vecchione G, Cappucci G, Giuliani N, Colaizzo D, Celentano E, Panico S, Di Minno G. Plasminogen activator inhibitor-1 (PAI-1) antigen plasma levels in subjects attending a metabolic ward: relation to polymorphisms of PAI-1 and angiontensin converting enzyme (ACE) genes. Arterioscler Thromb Vasc Biol. 1997;17(10):2082-7.

Saidi S, Slamia LB, Mahjoub T, Ammou SB, Almawi WY. Association of PAI-1 4G/5G and -844G/A gene polymorphism and changes in PAI-1/tPA levels in stroke: a case-control study. J Stroke Cerebrovasc Dis. 2007;16(4):153-9.

tPA Levels - Stroke

Carter AM, Catto AJ, Grant PJ. Determinants of tPA antigen and associations with coronary artery disease and acute cerebrovascular disease. Thromb Haemost. 1998;80(4):632-6.

Johansson L, Jansson JH, Boman K, Nilsson TK, Stegmayr B, Hallmans G. Tissue plasminogen activator, plasminogen activator inhibitor-1, and tissue plasminogen activator/plasminogen activator inhibitor-1 complex as risk factors for the development of a first stroke. Stroke. 2000;31(1):26-32.

Jood K, Ladenvall P, Tjarnlund-Wolf A, et al. Fibrinolytic gene polymorphism and ischemic stroke. Stroke. 2005;36(10):2077-81.

Kristensen B, Malm J, Nilsson TK, Hultdin J, Carlberg B, Olsson T. Increased fibrinogen levels and acquired hypofibrinolysis in young adults with ischemic stroke. Stroke. 1998;29(11):2261-7.

Macko RF, Kittner SJ, Epstein A, Cox DK, Wozniak MA, Wityk RJ, Stern BJ, Sloan MA, Sherwin R, Price TR, McCarter RJ, Johnson CJ, Earley CJ, Buchholz DW, Stolley PD. Elevated tissue plasminogen activator antigen and stroke risk: The Stroke Prevention In Young Women Study. Stroke. 1999;30(1):7-11.

Saidi S, Slamia LB, Mahjoub T, Ammou SB, Almawi WY. Association of PAI-1 4G/5G and -844G/A gene polymorphism and changes in PAI-1/tPA levels in stroke: a case-control study. J Stroke Cerebrovasc Dis. 2007;16(4):153-9.

Smith A, Patterson C, Yarnell J, Rumley A, Ben-Shlomo Y, Lowe G. Which hemostatic markers add to the predictive value of conventional risk factors for coronary heart disease and ischemic stroke? The Caerphilly Study. Circulation. 2005;112(20):3080-7.

Smith FB, Lee AJ, Fowkes FG, Price JF, Rumley A, Lowe GD. Hemostatic factors as predictors of ischemic heart disease and stroke in the Edinburgh Artery Study. Arterioscler Thromb Vasc Biol. 1997;17(11):3321-5.

Tzoulaki I, Murray GD, Lee AJ, Rumley A, Lowe GD, Fowkes FG. Relative value of inflammatory, hemostatic, and rheological factors for incident myocardial infarction and stroke: the Edinburgh Artery Study. Circulation. 2007;115(16):2119-27.

GLYCOPROTEIN IIIA

Carlsson LE, Greinacher A, Spitzer C, Walther R, Kessler C. Polymorphisms of the human platelet antigens HPA-1, HPA-2, HPA-3, and HPA-5 on the platelet receptors for fibrinogen (GPIIb/IIIa), von Willebrand factor (GPIb/IX), and collagen (GPIa/IIa) are not correlated with an increased risk for stroke. Stroke. 1997;28:1392-1395.

Carter AM, Catto AJ, Bamford JM, Grant PJ. Association of the platelet glycoprotein IIb HPA-3 polymorphism with survival after acute ischemic stroke. Stroke. 1999;30:2606-2611.

Corral J, González-Conejero R, Rivera J, Iniesta JA, Lozano ML, Vicente V. HPA-1 genotype in arterial thrombosis--role of HPA-1b polymorphism in platelet function. Blood Coagul Fibrinolysis. 1997 Jul;8(5):284-90.

Iniesta JA, Corral J, Gonzalez-Conejero R, Rivera J, Vicente V. Prothrombotic genetic risk factors in patients with coexisting migraine and ischemic cerebrovascular disease. Headache. 1999;39:486-489.

Kekomaki S, Hamalainen L, Kauppinen-Makelin R, Palomaki H, Kaste M, Kontula K. Genetic polymorphism of platelet glycoprotein IIIa in patients with acute myocardial infarction and acute ischaemic stroke. J Cardiovasc Risk. 1999;6:13-17.

Lanni F, Santulli G, Izzo R, et al. The Pl(A1/A2) polymorphism of glycoprotein IIIa and cerebrovascular events in hypertension: increased risk of ischemic stroke in high-risk patients. J Hypertens. 2007;25(3):551-6.

Reiner AP, Kumar PN, Schwartz SM, et al. Genetic variants of platelet glycoprotein receptors and risk of stroke in young women. Stroke. 2000;31:1628-1633.

Reuner KH, Elgas M, Kaps M, Ruf A, Patscheke H. The human platelet antigen HPA-1a/1b (PI(A1)/PI(A2)) polymorphism and cerebral ischaemia [letter]. Thromb Haemost. 1997;78:964-965.

Ridker PM, Hennekens CH, Schmitz C, Stampfer MJ, Lindpaintner K. PIA1/A2 polymorphism of platelet glycoprotein IIIa and risks of myocardial infarction, stroke, and venous thrombosis. Lancet. 1997;349:385-388.

Slowik A, Dziedzic T, Turaj W, et al. A2 alelle of GpIIIa gene is a risk factor for stroke caused by large-vessel disease in males. Stroke. 2004;35(7):1589-93

Streifler JY, Rosenberg N, Chetrit A, et al. Cerebrovascular events in patients with significant stenosis of the carotid artery are associated with hyperhomocysteinemia and platelet antigen-1 (Leu33Pro) polymorphism. Stroke. 2001 1;32(12):2753-8.

Szolnoki Z, Somogyvari F, Kondacs A, et al. Increased prevalence of platelet glycoprotein IIb/IIIa PLA2 allele in ischaemic stroke associated with large vessel pathology. Thromb Res. 2003;109(5-6):265-9.

van Goor ML, Gómez García E, Brouwers GJ, Leebeek FW, Koudstaal PJ, Dippel DW. PLA1/A2 polymorphism of the platelet glycoprotein receptor IIb/IIIa in young patients with cryptogenic TIA or ischemic stroke. Thrombosis Research. 2002;108:63-65

Wagner KR, Giles WH, Johnson CJ, et al. Platelet glycoprotein receptor IIIa polymorphism P1A2 and ischemic stroke risk: the Stroke Prevention in Young Women Study. Stroke. 1998;29:581-585.

PDE4D

Bevan S, Porteous L, Sitzer M, Markus HS. Phosphodiesterase 4D gene, ischemic stroke, and asymptomatic carotid atherosclerosis. Stroke. 2005;36(5):949-53.

Fidani L, Clarimon J, Goulas A, et al. Association of phosphodiesterase 4D gene G0 haplotype and ischaemic stroke in a Greek population. Eur J Neurol. 2007;14(7):745-9.

Gretarsdottir S, Thorleifsson G, Reynisdottir ST, et al. The gene encoding phosphodiesterase 4D confers risk of ischemic stroke. Nat Genet. 2003;35(2):131-8

Kostulas K, Gretarsdottir S, Kostulas V, et al. PDE4D and ALOX5AP genetic variants and risk for Ischemic Cerebrovascular Disease in Sweden. J Neurol Sci. 2007;263(1-2):113-7.

Kuhlenbaumer G, Berger K, Huge A, et al. Evaluation of single nucleotide polymorphisms in the phosphodiesterase 4D gene (PDE4D) and their association with ischaemic stroke in a large German cohort. J Neurol Neurosurg Psychiatry. 2006;77(4):521-4.

Lohmussaar E, Gschwendtner A, Mueller JC, et al. ALOX5AP gene and the PDE4D gene in a central European population of stroke patients. Stroke. 2005;36(4):731-6

Meschia JF, Brott TG, Brown RD Jr, et al. Phosphodiesterase 4D and 5-lipoxygenase activating protein in ischemic stroke. Ann Neurol. 2005;58(3):351-61

Nilsson-Ardnor S, Wiklund PG, Lindgren P, et al. Linkage of ischemic stroke to the PDE4D region on 5q in a Swedish population. Stroke. 2005;36(8):1666-71

Staton JM, Sayer MS, Hankey GJ, et al. Association between phosphodiesterase 4D gene and ischaemic stroke. J Neurol Neurosurg Psychiatry. 2006;77(9):1067-9.

van Rijn MJ, Slooter AJ, Schut AF, et al. Familial aggregation, the PDE4D gene, and ischemic stroke in a genetically isolated population. Neurology. 2005;65(8):1203-9.

Zee RY, Brophy VH, Cheng S, Hegener HH, Erlich HA, Ridker PM. Polymorphisms of the phosphodiesterase 4D, cAMP-specific (PDE4D) gene and risk of ischemic stroke: a prospective, nested case-control evaluation. Stroke. 2006;37(8):2012-7.

APOLIPOPROTEIN E

Abboud S, Viiri LE, Lütjohann D, Goebeler S, Luoto T, Friedrichs S, Desfontaines P, Gazagnes MD, Laloux P, Peeters A, Seeldrayers P, Lehtimaki T, Karhunen P, Pandolfo M, Laaksonen R. Associations of apolipoprotein E gene with ischemic stroke and intracranial atherosclerosis. Eur J Hum Genet. 2008;16(8):955-60.

Basun H, Corder EH, Guo Z, et al. Apolipoprotein E polymorphism and stroke in a population sample aged 75 years or more. Stroke. 1996;27:1310-1315.

Catto AJ, McCormack LJ, Mansfield MW, et al. Apolipoprotein E polymorphism in cerebrovascular disease. Acta Neurol Scand. 2000;101:399-404.

Cerrato P, Baima C, Grasso M, et al. Apolipoprotein E polymorphism and stroke subtypes in an Italian cohort. Cerebrovasc Dis. 2005;20(4):264-9.

Couderc R, Mahieux F, Bailleul S, Fenelon G, Mary R, Fermanian J. Prevalence of apolipoprotein E phenotypes in ischemic cerebrovascular disease: a case-control study. Stroke. 1993;24:661-664.

Duzenli S, Pirim I, Gepdiremen A, Deniz O. Apolipoprotein E polymorphism and stroke in a population from eastern Turkey. J Neurogenet. 2004;18(1):365-75

Frikke-Schmidt R, Nordestgaard BG, Thudium D, Moes Gronholdt ML, Tybjaerg-Hansen A. APOE genotype predicts AD and other dementia but not ischemic cerebrovascular disease. Neurology. 2001;56:194-200.

Hachinski V, Graffagnino C, Beaudry M, et al. Lipids and stroke: a paradox resolved. Arch Neurol. 1996;53:303-308.

Karttunen V, Alfthan G, Hiltunen L, et al. Risk factors for cryptogenic ischaemic stroke. Eur J Neurol. 2002;9(6):625-32.

Kessler C, Spitzer C, Stauske D, et al. The apolipoprotein E and β-fibrinogen G/A-455 gene polymorphisms are associated with ischemic stroke involving large-vessel disease. Arterioscler Thromb Vasc Biol. 1997;17:2880-2884.

MacLeod MJ, De Lange RP, Breen G, Meiklejohn D, Lemmon H, Clair DS. Lack of association between apolipoprotein E genotype and ischaemic stroke in a Scottish population. Eur J Clin Invest. 2001;31:570-573.

Margaglione M, Seripa D, Gravina C, et al. Prevalence of apolipoprotein E alleles in healthy subjects and survivors of ischemic stroke: an Italian Case-Control Study. Stroke. 1998;29:399-403.

McIlroy SP, Dynan KB, Lawson JT, Patterson CC, Passmore AP. Moderately elevated plasma homocysteine, methylenetetrahydrofolate reductase genotype, and risk for stroke, vascular dementia, and Alzheimer disease in Northern Ireland. Stroke. 2002;33:2351-2356.

Pezzini A, Grassi M, Del Zotto E, et al. Cumulative effect of predisposing genotypes and their interaction with modifiable factors on the risk of ischemic stroke in young adults. Stroke. 2005;36(3):533-9

Saidi S, Slamia LB, Mahjoub T, Ammou SB, Almawi WY. Association of PAI-1 4G/5G and -844G/A gene polymorphism and changes in PAI-1/tPA levels in stroke: a case-control study. J Stroke Cerebrovasc Dis. 2007;16(4):153-9.

Slowik A, Iskra T, Turaj W, Hartwich J, Dembinska-Kiec A, Szczudlik A. LDL phenotype B and other lipid abnormalities in patients with large vessel disease and small vessel disease. J Neurol Sci. 2003;214(1-2):11-6

Tasdemir N, Tamam Y, Toprak R, Tamam B, Tasdemir MS. Association of apolipoprotein E genotype and cerebrovascular disease risk factors in a Turkish population. Int J Neurosci. 2008;118(8):1109-29.

Topic E, Timundic AM, Ttefanovic M, et al. Polymorphism of apoprotein E (APOE), methylenetetrahydrofolate reductase (MTHFR) and paraoxonase (PON1) genes in patients with cerebrovascular disease. Clin Chem Lab Med. 2001;39:346-350.

Traykov L, Bayle AC, Latour F, et al. Apolipoprotein E epsilon4 allele frequency in elderly depressed patients with and without cerebrovascular disease. J Neurol Sci. 2007;257(1-2):280-3.

FACTOR XIII

Catto AJ, Kohler HP, Bannan S, Stickland M, Carter A, Grant PJ. Factor XIII Val 34 Leu: a novel association with primary intracerebral hemorrhage. Stroke. 1998;29:813-816.

Corral J, Gonzalez-Conejero R, Iniesta JA, Rivera J, Martinez C, Vicente V. The FXIII Val34Leu polymorphism in venous and arterial thromboembolism. Haematologica. 2000;85:293-297.

Elbaz A, Poirier O, Canaple S, Chedru F, Cambien F, Amarenco P. The association between the Val34Leu polymorphism in the factor XIII gene and brain infarction. Blood. 2000;95:586-591.

Endler G, Funk M, Haering D, et al. Is the factor XIII 34Val/Leu polymorphism a protective factor for cerebrovascular disease? Br J Haematol. 2003;120:310-314.

Gemmati D, Serino ML, Ongaro A, et al. A common mutation in the gene for coagulation factor XIII-A (VAL34Leu): a risk factor for primary intracerebral hemorrhage is protective against atherothrombotic diseases. Am J Hematol. 2001;67:183-188.

Reiner AP, Frank MB, Schwartz SM, et al. Coagulation factor XIII polymorphisms and the risk of myocardial infarction and ischaemic stroke in young women. Br J Haematol. 2002;116:376-382.

Rubattu S, Di Angelantonio E, Nitsch D, et al. Polymorphisms in prothrombotic genes and their impact on ischemic stroke in a Sardinian population. Thromb Haemost. 2005;93(6):1095-100

Slowik A, Dziedzic T, Pera J, Figlewicz DA, Szczudlik A. Coagulation factor XIII VaI34Leu polymorphism in patients with small vessel disease or primary intracerebral hemorrhage. Cerebrovasc Dis. 2005;19(3):165-70

ANGIOTENSINOGEN

Barley J, Markus H, Brown M, Carter N. Lack of association between angiotensinogen polymorphism (M235T) and cerebrovascular disease and carotid atheroma. J Hum Hypertens. 1995;9(8):681-3.

Bis JC, Smith NL, Psaty BM, et al. Angiotensinogen Met235Thr polymorphism, angiotensin-converting enzyme inhibitor therapy, and the risk of nonfatal stroke or myocardial infarction in hypertensive patients. Am J Hypertens. 2003;16(12):1011-7

Brenner D, Labreuche J, Poirier O, Cambien F, Amarenco P; GENIC Investigators. Renin-angiotensin-aldosterone system in brain infarction and vascular death. Ann Neurol. 2005;58(1):131-8

Gormley K, Bevan S, Markus HS. Polymorphisms in genes of the renin-angiotensin system and cerebral small vessel disease. Cerebrovasc Dis. 2007;23(2-3):148-55.

Sethi AA, Tybjaerg-Hansen A, Grønholdt ML, Steffensen R, Schnohr P, Nordestgaard BG. Angiotensinogen mutations and risk for ischemic heart disease, myocardial infarction, and ischemic cerebrovascular disease. Six case-control studies from the Copenhagen City Heart Study. Ann Intern Med. 2001 May 15;134(10):941-54.

GLYCOPROTEIN 1balpha – KOZAK

Baker RI, Eikelboom J, Lofthouse E, et al. Platelet glycoprotein Ibα Kozak polymorphism is associated with an increased risk of ischemic stroke. Blood. 2001;98:36-40.

Carter AM, Catto AJ, Bamford JM, Grant PJ. Platelet GP IIIa PlA and GP Ib variable number tandem repeat polymorphisms and markers of platelet activation in acute stroke. Arterioscler Thromb Vasc Biol. 1998;18:1124-1131

Corral J, Lozano ML, Gonzalez-Conejero R, et al. A common polymorphism flanking the ATG initiator codon of GPIb alpha does not affect expression and is not a major risk factor for arterial thrombosis. Thromb Haemost. 2000;83:23-28

Frank MB, Reiner AP, Schwartz SM, et al. The Kozak sequence polymorphism of platelet glycoprotein Ibα and risk of nonfatal myocardial infarction and nonfatal stroke in young women. Blood. 2001;97:875-879.

Hsieh K, Funk M, Schillinger M, et al. Vienna Stroke Registry. Impact of the platelet glycoprotein Ib alpha Kozak polymorphism on the risk of ischemic cerebrovascular events: a case-control study. Blood Coagul Fibrinolysis. 2004;15(6):469-73

eNOS

Elbaz A, Poirier O, Moulin T, et al. Association between the Glu298Asp polymorphism in the endothelial constitutive nitric oxide synthase gene and brain infarction. Stroke. 2000;31:1634-1639.

Guldiken B, Sipahi T, Guldiken S, Ustundag S, Budak M, Turgut N, Ozkan H. Glu298Asp polymorphism of the endothelial nitric oxide synthase gene in Turkish patients with ischemic stroke. Mol Biol Rep. 2009;36(6):1539-43.

Hassan A, Gormley K, O'Sullivan M, et al. Endothelial nitric oxide gene haplotypes and risk of cerebral small-vessel disease. Stroke. 2004;35(3):654-9

MacLeod MJ, Dahiyat MT, Cumming A, Meiklejohn D, Shaw D, St Clair D. No association between Glu/Asp polymorphism of NOS3 gene and ischemic stroke. Neurology. 1999;53:418-420.

Markus HS, Ruigrok Y, Ali N, Powell JF. Endothelial nitric oxide synthase exon 7 polymorphism, ischemic cerebrovascular disease, and carotid atheroma. Stroke. 1998;29:1908-1911.

INTERLEUKIN 6

Balding J, Livingstone WJ, Pittock SJ, et al. The IL-6 G-174C polymorphism may be associated with ischaemic stroke in patients without a history of hypertension. Ir J Med Sci 2004; 173 (4): 200-3

Chamorro A, Revilla M, Obach V, Vargas M, Planas AM. The -174G/C polymorphism of the interleukin 6 gene is a hallmark of lacunar stroke and not other ischemic stroke phenotypes. Cerebrovasc Dis. 2005;19(2):91-5

Flex A, Gaetani E, Papaleo P, et al. Proinflammatory genetic profiles in subjects with history of ischemic stroke. Stroke. 2004;35(10):2270-5.

Greisenegger S, Endler G, Haering D, et al. The (-174) G/C polymorphism in the interleukin-6 gene is associated with the severity of acute cerebrovascular events. Thromb Res. 2003;110(4):181-6

Lalouschek W, Schillinger M, Hsieh K, et al. Polymorphisms of the inflammatory system and risk of ischemic cerebrovascular events. Clin Chem Lab Med. 2006;44(8):918-23.

Pola R, Flex A, Gaetani E, Flore R, Serricchio M, Pola P. Synergistic effect of -174 G/C polymorphism of the interleukin-6 gene promoter and 469 E/K polymorphism of the intercellular adhesion molecule-1 gene in Italian patients with history of ischemic stroke. Stroke. 2003;34(4):881-5

Revilla M, Obach V, Cervera A, Dávalos A, Castillo J, Chamorro A. A -174G/C polymorphism of the interleukin-6 gene in patients with lacunar infarction. Neurosci Lett. 2002; 324(1):29-32.

HFE

Ekblom K, Hultdin J, Stegmayr B, et al. Iron stores and HFE genotypes are not related to increased risk of ischemic stroke. A prospective nested case-referent study. Cerebrovasc Dis. 2007;24(5):405-11.

Ellervik C, Tybjaerg-Hansen A, Appleyard M, Sillesen H, Boysen G, Nordestgaard BG. Hereditary hemochromatosis genotypes and risk of ischemic stroke. Neurology. 2007;68(13):1025-31.

Hetet G, Elbaz A, Gariepy J, et al. Association studies between haemochromatosis gene mutations and the risk of cardiovascular diseases. Eur J Clin Invest. 2001;31(5):382-8.

Hruskovicova H, Milanez T, Kobal J, Potisk KP, Petrovic D, Peterlin B. Hemochromatosis-causing mutations C282Y and H63D are not risk factors for atherothrombotic cerebral infarction. Med Sci Monit. 2005;11(7):248-52

Njajou OT, Hollander M, Koudstaal PJ, et al. Mutations in the hemochromatosis gene (HFE) and stroke. Stroke. 2002;33(10):2363-6

ANGIOTENSIN RECEPTOR

Brenner D, Labreuche J, Poirier O, Cambien F, Amarenco P; GENIC Investigators. Renin-angiotensin-aldosterone system in brain infarction and vascular death. Ann Neurol. 2005;58(1):131-8

Möllsten A, Stegmayr B, Wiklund PG. Genetic polymorphisms in the renin-angiotensin system confer increased risk of stroke independently of blood pressure: a nested case-control study. J Hypertens. 2008;26(7):1367-72.

Rubattu S, Di Angelantonio E, Stanzione R, Zanda B, Evangelista A, Pirisi A, De Paolis P, Cota L, Brunetti E, Volpe M. Gene polymorphisms of the renin-angiotensin-aldosterone system and the risk of ischemic stroke: a role of the A1166C/AT1 gene variant. J Hypertens. 2004;22(11):2129-34.

Szolnoki Z, Maasz A, Magyari L, et al. Coexistence of angiotensin II type-1 receptor A1166C and angiotensin-converting enzyme D/D polymorphism suggests susceptibility for small-vessel-associated ischemic stroke. Neuromolecular Med. 2006;8(3):353-60.

FACTOR VII

Corral J, Gonzales-Conejero R, Lozano ML, Rivera J, Vicente V. Genetic polymorphisms of factor VII are not associated with arterial thrombosis. Blood Coagul Fibrinolysis. 1998;9:267-272

Heywood DM, Carter AM, Catto AJ, Bamford JM, Grant PJ. Polymorphisms of the factor VII gene and circulating FVII:C levels in relation to acute cerebrovascular disease and poststroke mortality. Stroke. 1997;28:816-821.

Petrovic D, Milanez T, Kobal J, Bregar D, Potisk KP, Peterlin B. Prothrombotic gene polymorphisms and atherothrombotic cerebral infarction. Acta Neurol Scand. 2003;108(2):109-13

Rubattu S, Di Angelantonio E, Nitsch D, et al. Polymorphisms in prothrombotic genes and their impact on ischemic stroke in a Sardinian population. Thromb Haemost. 2005;93(6):1095-100

Zee RY, Cook NR, Cheng S, Reynolds R, Erlich HA, Lindpaintner K, Ridker PM. Polymorphism in the P-selectin and interleukin-4 genes as determinants of stroke: a population-based, prospective genetic analysis. Hum Mol Genet. 2004;13(4):389-96.
